# Achieving 20% Toluene-Processed Binary Organic Solar Cells via Secondary Regulation of Donor Aggregation in Sequential Processing

**DOI:** 10.1007/s40820-025-01715-2

**Published:** 2025-04-01

**Authors:** Yufei Wang, Chuanlin Gao, Wen Lei, Tao Yang, Zezhou Liang, Kangbo Sun, Chaoyue Zhao, Lu Chen, Liangxiang Zhu, Haoxuan Zeng, Xiaokang Sun, Bin He, Hanlin Hu, Zeguo Tang, Mingxia Qiu, Shunpu Li, Peigang Han, Guangye Zhang

**Affiliations:** 1 College of New Materials and New Energies, Shenzhen Technology University, Shenzhen, 518118 People’s Republic of China; 2 College of Cyber Security, Jinan University, Guangzhou, 511443 People’s Republic of China; 3 Future Technology School, Shenzhen Technology University, Shenzhen, 518118 People’s Republic of China; 4 Key Laboratory for Physical Electronics and Devices of the Ministry of Education and Shaanxi Key Lab of Photonic, Technique for Information, School of Electronics Science and Engineering, Faculty of Electronic and Information Engineering, Xi’an Jiaotong University, Xi’an, 710049 People’s Republic of China; 5 Hoffmann Institute of Advanced Materials, Shenzhen Polytechnic University, Shenzhen, 518055 People’s Republic of China

**Keywords:** Organic solar cells, Non-halogen solvent, Sequential processing, Secondary nucleation, Stability

## Abstract

Sequential processing (SqP) of the active layer offers independent optimization of the donor and acceptor with more targeted solvent design, which is considered the most promising strategy for achieving efficient organic solar cells (OSCs). In the SqP method, the favorable interpenetrating network seriously depends on the fine control of the bottom layer swelling. However, the choice of solvent(s) for both the donor and acceptor have been mostly based on a trial-and-error manner. A single solvent often cannot achieve sufficient yet not excessive swelling, which has long been a difficulty in the high efficient SqP OSCs. Herein, two new isomeric molecules are introduced to fine-tune the nucleation and crystallization dynamics that allows judicious control over the swelling of the bottom layer. The strong non-covalent interaction between the isomeric molecule and active materials provides an excellent driving force for optimize the swelling-process. Among them, the molecule with high dipole moment promotes earlier nucleation of the PM6 and provides extended time for crystallization during SqP, improving bulk morphology and vertical phase segregation. As a result, champion efficiencies of 17.38% and 20.00% (certified 19.70%) are achieved based on PM6/PYF-T-*o* (all-polymer) and PM6/BTP-eC9 devices casted by toluene solvent.

**Supplementary Information:**

The online version contains supplementary material available at 10.1007/s40820-025-01715-2.

## Highlights

- Isomeric molecules are introduced to fine-tune the secondary nucleation process of the donor underlayer.
- Binary organic solar cell processed with non-halogen solvent shows a champion efficiency of 20.0% (certified 19.7%).
- Mechanism of the film formation processes and the sequential processing community about solvent design rules are proposed.

**Supplementary Information:**

The online version contains supplementary material available at 10.1007/s40820-025-01715-2.

## Graphical abstract

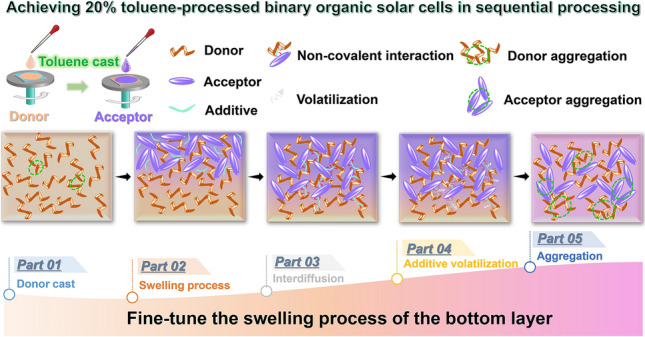

## Introduction

Organic solar cells (OSCs) have been extensively studied owing to the charming features of light-weight, flexibility, semitransparency, compatibility with roll-to-roll solution-based production, etc. [1–6]. Non-fullerene acceptors with high absorption coefficients in the red and near-infrared region has significantly improved of the performance of OSCs [7–10]. The combined effort of materials innovation and device engineering leads to the ~ 20% power conversion efficiency (PCE) of single-junction OSCs [11–13]. However, a high fraction of the top-of-the-line PCEs are realized through the use of highly vaporizable solvents such as chloroform to ‘lock’ the morphology of the active layer in a relatively instable thermodynamic non-equilibrium state. Along with its unneglectable toxicity, the potential of chloroform in large-scale production is very limited [14, 15]. In this context, employing greener solvents with a high-boiling point to achieve high efficiency OSCs is essential for the industrialization of the technology. However, in order to balance charge transport and film morphology, the state-of-the-art OSCs are composed of polymer donors and non-fullerene acceptors with a complex fused-ring framework and lengthy side chains, which typically results in a low solubility in most non-halogenated solvents [15, 16]. Without extra post-treatments, the aggregation, crystallization and phase segregation of the active layer blend casted by non-halogenated solvents, strongly affected by the drying kinetics, are difficult to regulate. The overall nanoscale morphology produced this way is typically in an unfavorable state, resulting in much lower efficiency than chloroform-casting devices [17–20]. Therefore, it is crucial to broaden the methods that can effectively control the morphology of active layer when high-boiling point non-halogen solvents are used to prepare the active layer. However, it is difficult to effectively adjust the molecular stacking and donor–acceptor phase segregation through targeted solvent-material interaction in a conventional blend-cast device, i.e., making the solvent selectively interact with the donor (or acceptor) alone.

This makes the sequential processing (SqP) approach even more important. SqP, also known as layer-by-layer (LBL) deposition, is the other mainstream active layer preparation technique of OSCs, which was first demonstrated by Schwartz et al. [21] whose basic mechanism was gradually revealed in 2011–2014 [22–24]. The SqP method prepares the donor and acceptor from independent solutions so it offers the opportunity for donor or acceptor to interact with specific solvent(s) alone prior to the coating process [21, 25–28]. This could allow judicious solvent and additive design without concerning the other material prior to film formation, which can better release the potential of solvent engineering in new materials [29–31]. However, despite a few attempts including our recent works [11, 25, 26, 28], targeted solvent and additive design has rarely been reported in SqP particularly when high-boiling point solvents are utilized. The choice of solvent(s) for both the donor and acceptor along with the place, where the additive is used (donor or acceptor solution), have been mostly based on a trial-and-error manner. The main issue is the morphology formation mechanism is not understood when new solvent(s) or additives are employed.

In the SqP method, the interpenetrating network between the donor and acceptor is formed by the swelling of the bottom polymer donor by the upper layer solution or by the residue solvent(s) in the underlayer, which is the main factor determining materials interdiffusion during film formation, phase separation and final photovoltaic performance in OSCs [24]. Therefore, developing a suitable and simple method to control the interdiffusion between upper acceptor and bottom polymer donor and thus control the morphology is particularly crucial for achieving efficient OSCs casted by non-halogen solvents. As the SqP method becomes more and more popular in recent high-performance systems, targeted solvent design with different solvent additives for different materials/layers is still strongly needed to fine-tune the crystallization kinetics of upper acceptor and drying process of the final film in OSCs prepared from non-halogen solvents, not only for the demonstration purpose that the SqP method has this unique advantage, but also for the further improvement of device performance.

In this work, we achieve SqP devices with 20% PCEs using toluene (high-boiling point hydrocarbon solvent) as the main processing solvent for both the upper and bottom layers through targeted additive-material interaction: Two novel additives (ODBC and PDBC) through an isomerization strategy are introduced to the SqP method to address the swelling and morphology issues in conventional toluene-based processing. These molecules can fine-tune the nucleation and drying kinetics of the donor and acceptor by the non-covalent interaction, which is key parameter to regulating the swelling process in the SqP device. Importantly, we show that introducing additives in the upper acceptor layer can endow the final active layer with better spectral response and stronger molecular aggregation than adding them in the bottom donor layer, which contributes to the prominent improvement of the photocurrent. Furthermore, the isomeric molecules induce an earlier nucleation of the PM6 compared with the control condition, which can significantly extend the drying time of the active film, providing a longer crystal coherence length and tighter π-π stacking. The strong non-covalent interaction between isomeric molecules and active molecules can enhance the infiltration between upper donors and bottom acceptors, endowing an optimal PM6 content near hole transport layer and an improved ultrafast exciton dissociation. Thus, an outstanding *J*_SC_ of 26.2 mA cm^−2^ is obtained in PM6/PYF-T-*o*:ODBC, which increases the PCE for the all-polymer solar cell from 14.88% to 17.38%. Moreover, the ODBC promotes PM6/BTP-eC9 based SqP device performance as well when toluene is used as the main processing solvent. The PCE reaches 18.5% for PEDOT:PSS based device and 20.0% for 2PACZ based device (19.7% certified). This work proves the importance of non-covalent interaction in regulating the swelling process of SqP, and highlights the potential of the SqP method combined with additive isomerization strategy in enhancing the performance of non-halogen solvent processed OSCs.

## Experimental Section

### Materials

PM6 was obtained from Solarmer Materials Inc., while PYF-T-*o*, BTP-eC9, and PNDIT-F3N were sourced from eFlexPV Limited. PEDOT:PSS (Clevios P VP 4083) was procured from Heraeus Inc., Germany. 2PACZ was acquired from TCI. All other reagents and chemicals were purchased from Sigma-Aldrich or Aladdin and used without further purification.

### Device Fabrication

The OSCs with the structure of ITO/PEDOT:PSS(4083) or 2PACZ/PM6/PYF-T-*o*/PNDIT-F3N/Ag were fabricated. The indium tin oxide (ITO) glass substrates were sequentially cleaned with acetone, detergent, deionized water, and ethanol in turn. Following this, the ITO substrates were subjected to oxygen plasma treatment for 5 min. A thin film of PEDOT:PSS (30 nm) was then spin-casted on the ITO substrates amd annealed at 160 °C for 15 min. For 2PACZ-based device, a solution of 2PACZ in methanol (0.3 mg mL^−1^) was prepared. The 2PACZ layer was spin-casted onto the ITO substrates at 3000 rpm and annealed at 100 °C for 5 min.

#### PM6:Additive/PYF-T-*o* Film Preparation

PM6 (8 mg mL^−1^), PM6:ODBC (donor concentration: 8 mg mL^−1^; weight ratios of 1:4, 1:6), PM6:PDBC (donor concentration: 8 mg mL^−1^; weight ratios of 1:6.25, 1:12.5, 1:18.75), and PYF-T-*o* (12 mg mL^−1^) were individually dissolved in toluene. The solutions were then stirred at 80 °C for 1 h. Subsequently, the PM6:ODBC or PM6:PDBC layers was deposited onto the PEDOT:PSS layer via spin-coating method (4000 rpm, 30 s). Next, the PYF-T-*o* layer was spin-casted on the PM6 layers at 3400 rpm for 30 s. Then, the resulting PM6:additive/PYF-T-*o* films were thermal annealing (80, 100, and 120 °C) for 5 min.

#### PM6/PYF-T-*o*:Additive Film Preparation

PM6 (8 mg mL^−1^) and PYF-T-*o* (12 mg mL^−1^) were dissolved in toluene. Additionally, PYF-T-*o*:ODBC and PYF-T-*o*:PDBC acceptor concentration (12 mg mL^−1^) at ratios of 1:2.7, 1:4.1, 1:5.5 (wt/wt), and 1:4.2, 1:8.3, 1:12.5 (wt/wt), respectively, were prepared in the same solvent. All solutions were stirred at 80 °C for 1 h. A PM6 layer was then spin-coated onto the PEDOT:PSS at 4000 rpm for 30 s, followed by the deposition of a PYF-T-*o*:additive layer at 3400 rpm for 30 s. Then, the resulting films underwent thermal annealing at 80, 100, or 120 °C for 5 min.

#### PM6:PYF-T-*o*:ODBC Film Preparation

Solutions of PM6:PYF-T-*o*:ODBC with donor concentration of 8 mg mL^−1^ and varying ODBC ratios (1:1.2:0, 1:1.2:1, 1:1.2:2, 1:1.2:4, 1:1.2:5 and 1:1.2:6 wt/wt) were dissolved in chloroform and stirred at 50 °C for 1 h. Films were formed via spin-coating at 2400 rpm for 30 s on PEDOT:PSS substrates, followed by thermal annealing at 80, 100, or 120 °C for 5 min.

#### PM6:ODBC/PYF-T-*o*:ODBC Film Preparation

PM6:ODBC (donor concentration 8 mg mL^−1^; ratios 1:1, 1:2, 1:4, 1:6 wt/wt), PYF-T-*o*:ODBC (acceptor concentration 8 mg mL^−1^; ratios 1.5:6 wt/wt) were dissolved in toluene and heated at 80 °C for 1 h. Afterward, layers were sequentially deposited using spin-coating at 4000 and 3400 rpm for PM6:ODBC and PYF-T-*o*:ODBC, respectively. Thermal annealing was performed at 100 °C for 5 min.

#### PM6/L8-BO:Additive Film Preparation

PM6 (8 mg mL^−1^), L8-BO (9 mg mL^−1^) and L8-BO:ODBC (acceptor concentration 8 mg mL^−1^; ratios 1:1.5 wt/wt) were dissolved in toluene and stirred at 70 °C for 1 h. Layers were spin-coated at 3400 rpm, with thermal annealing conducted at 80, 100, 120 °C for 5 min.

#### PM6/BTP-eC9:Additive Film Preparation

PM6 (8 mg mL^−1^), BTP-eC9 (8 mg mL^−1^) and BTP-eC9:ODBC (acceptor concentration 8 mg mL^−1^; ratio 1:2 wt/wt) were prepared in toluene and stirred at 70 °C for 1 h. Spin-coating was performed at 3800 rpm for PM6 and 3500 rpm for BTP-eC9:additives,followed by thermal annealing at 90 °C for 5 min.

#### PM6/PJ1-*γ*:Additive Film Preparation

PM6 (8 mg mL^−1^), PJ1-*γ* (10 mg mL^−1^), PJ1-*γ*:ODBC (acceptor concentration, 10 mg mL^−1^; ratio 1:3, wt/wt), and PJ1-*γ*:PDBC (acceptor concentration 10 mg mL^−1^; ratio 1:5, wt/wt) were individually dissolved in toluene. The PM6 layer was then spin-coated onto the PEDOT:PSS at 2800 rpm. Subsequently, the PJ1-*γ*:additive layer was deposited on top at 3800 rpm for 30 s. The resulting PM6/PJ1-*γ*:additive films underwent annealing at 100 °C for 5 min.

For all devices, a PNDIT-F3N solution (0.5 mg mL^−1^ in methanol containing 0.5 v/v acetic acid) was spin-coated onto the active layer at 2000 rpm for 30 s. Finally, approximately 100 nm of Ag was evaporated under 1 × 10^–4^ Pa through a shadow mask (aperture area: 3.95 mm^2^), encapsulation was subsequently performed.

## Results and Discussion

### Effect Mechanism of Additives on the Film Formations

Figure S1 shows the chemical structures of photoactive layer and solvents. Herein, we choose PM6 as the electron donor and a polymeric small molecule acceptor, PYF-T-*o*, as the electron acceptor (Fig. S1). The main solvent for the whole SqP process is toluene, along with two positional isomerized molecules serving as solvent or solid additives as shown in Fig. S1 and Table S1. The SqP technique is used for fabricating the OSCs [26]. The absorption spectra of the two isomerization additives is ranging from 280 to 350 nm (Fig. S2), which means that their introduction will hardly affect the capture of incident light by the active layer. The two isomeric additives exhibit a similar boiling point (196–204 °C), both higher than that of the main solvent, toluene (110.6 °C), which may can provide a long film drying time to optimize the mutual permeation between the bottom donor layer and upper acceptor layer in the SqP device. At room temperature, ODBC (melting point in the range of − 13 to 11 °C) and PDBC (melting point ~ 64 °C) are liquid additive and solid additive, respectively, which is influenced by the position of bromine and chlorine substitution on the benzene ring [32]. Both ODBC and PDBC show an extremely low volatility temperature (below 120 °C) based on the thermogravimetric analysis curves (Fig. S3), which also shows that they can be removed without any residue when the temperature reaches about 200 °C. The 5% weight loss temperature (*T*_5% weight loss_) and complete volatilization temperature (*T*_complete volatilization_) of ODBC are 114 and 189 °C, respectively, slightly higher than those of PDBC (103 and 174 °C, respectively), which indicates that the tendency of vaporization for ODBC is weaker. The X-ray photoelectron spectra (XPS) of the PM6/PYF-T-*o*:ODBC or PDBC films before and after thermal annealing are further measured to evaluate the volatility of the additives (Fig. S4). The unique bromine and chlorine signals are used to detect additives in the active layer film. The intensity of Br 3*d* and Cl 2*p* signals near the surface of the PM6/PYF-T-*o*:ODBC and PM6/PYF-T-*o*:PDBC films with or without thermal annealing (100 °C, 5 min) are similar to the control film of PM6/PYF-T-*o*. In addition, the photos of ODBC and PDBC additive under different spin-coating times are also taken (Fig. S5). Furthermore, we also upload videos (Movie S1) to show the difference in the film drying between the two additive-involved spin-coating processes. The above results indicate that the volatilization process of two isomeric additives occur and complete during the spin-coating process without the need for extra post-processing treatments, which reduces the morphology change and thus in the later device fabrication steps and is thus beneficial for enhancing reproducibility and stability.

**Fig. 1:**
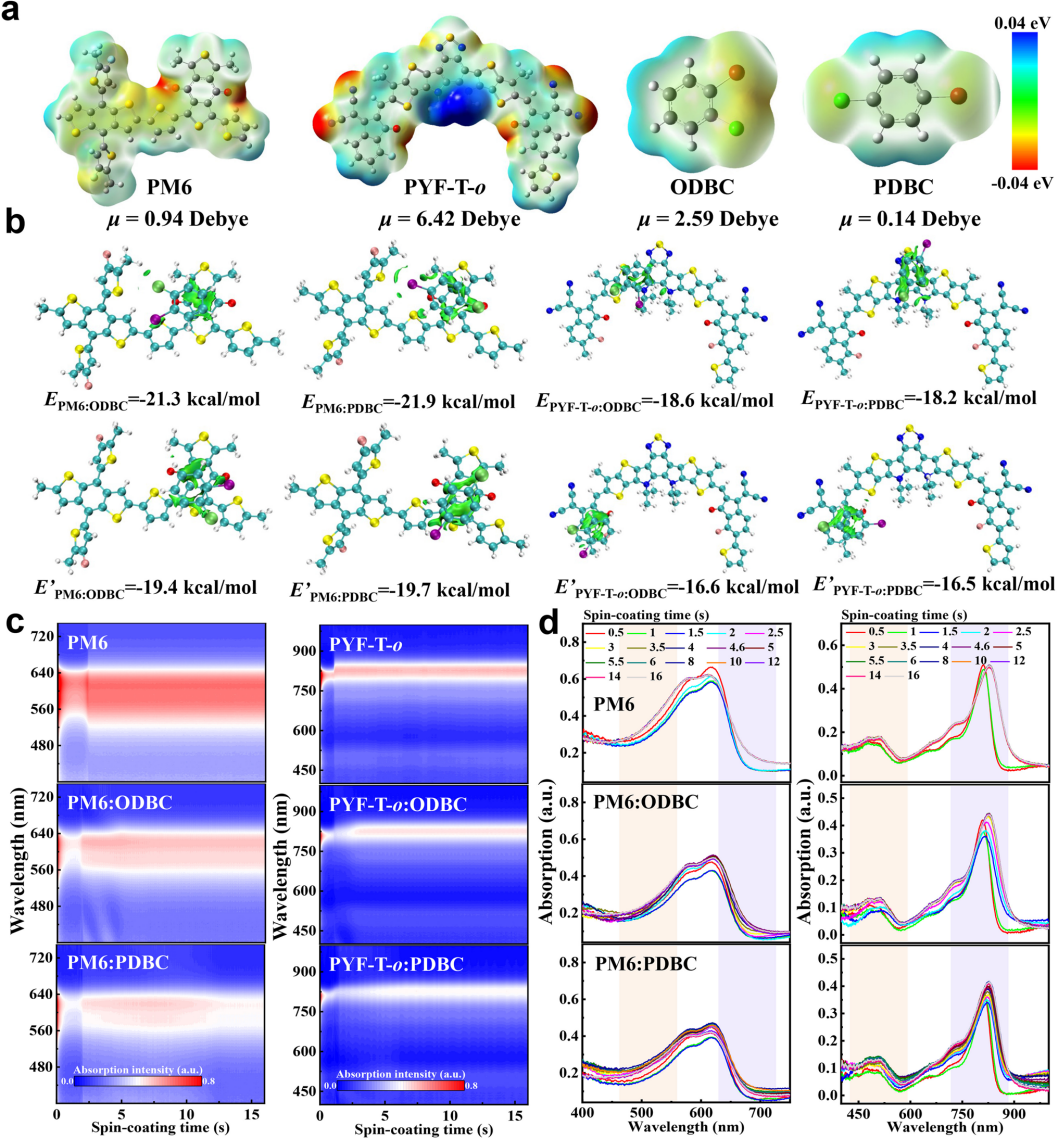
**a** ESP distribution and dipole moments of PM6, PYF-T-*o* and the two isomeric additives. **b** Calculated non-covalent interaction energies between PM6 or PYF-T-*o* and the isomeric additives under different adsorption sites. **c** 2D *in-situ* absorption spectra of PM6:additive and PYF-T-*o*:additive from solution to film state measured during spin-coating. **d** Absorption spectra at representative times during or after spin-coating

Next, we analyze the molecular interaction between additives and photoactive materials by density functional theory (DFT, Fig. 1a, b). To simplify the calculation process, the monomers of PM6 and PYF-T-*o* are selected, and the long alkyl side chains in their chemical structures are ignored. The electrostatic potential (ESP) distribution is firstly used to analyze the possible adsorption sites between additives and active molecules (Fig. 1a). In PM6, negative charge density is high around the sulfur and oxygen atoms, while higher positive charge density is found in the central BTP core in PYF-T-*o*. Meanwhile, we also observe some sporadic negative charge density near the F atoms and carbonyl/cyano groups. In antipolar attraction theory, the regions with more negative charge distribution in two isomeric additives around the halogen atoms (Br and Cl) can produce a coulomb attraction with the highly positive region in PYF-T-*o*. In contrast, the positively unsubstituted carbon atoms on the benzene ring in ODBC or PDBC can form another coulomb attraction with oxygen atoms in PM6. Therefore, ODBC and PDBC can form strong or weak non-covalent interaction with the PM6 or PYF-T-*o*, which may play a critical role in regulating the aggregation behaviors of donor and acceptor. In particular, the non-covalent interaction between additive and active materials is crucial in tuning the swelling process of the underlayer in the SqP method. Next, the non-covalent interaction calculations between additive and PM6 or PYF-T-*o* under different absorption sites based on B3LYP-(D3)BJ/def2-SVP level (Fig. 1b). The non-covalent interaction between PM6 and the isomeric additives with binding energies (*E*_PM6:additive_) in the range of −19.4  to  −21.9 kcal mol^−1^ is higher than that between PYF-T-*o* and additives (*E*_PYF-T-*o*:additive_ = -16.5 ~ -18.6 kcal mol^−1^). Under the same the adsorption site, the values of *E*_PM6:ODBC_ and *E*_PYF-T-*o*:ODBC_ are similar to *E*_PM6:PDBC_ and *E*_PYF-T-*o*:PDBC_, respectively. However, the solvent additive ODBC shows a higher dipole moment (*μ* = 2.59 Debye), which should increase its intermolecular interaction with PM6 or PYF-T-*o*.

Then, the effects of solvent and solid additives on the phase transition process (from solution to solid state) in pristine PM6 or PYF-T-*o* films are explored by in-situ absorption spectra during spin-coating (Fig. 1c). Representative absorption spectra of these sample films are shown in Fig. 1d, while the position and absorption intensity of the PM6_0-0_ or PYF-T-*o*_0-0_ peaks during spin-coating are shown in Fig. S6. The whole film formation process can be divided into three different stages for all sample films: (1) quick solvent evaporation with rapidly decreased absorption intensity; (2) crystallization/aggregation process with peak position shifting; and (3) formation of the solid film [33, 34]. It can be seen that the usage of ODBC or PDBC can extend the drying time of PM6 film from ~ 2.5 s to over 8 s (Fig. S5), which is resulted from their higher boiling points (196–204 °C) than the main solvent, toluene (110.6 °C) [35]. During 2–10 s, a redshifted position and a reduced absorption intensity of PM6_0-0_ peak are simultaneously found in sample films processed with additives compared with the control condition, indicative of a strong *J*-aggregation with stronger molecular stacking, which can be attributed to the strong non-covalent interaction (− 19.4 to − 21.9 kcal mol^−1^) between ODBC or PDBC and PM6. The *J*-aggregation induced by the solid additive exhibits a decay over spin-coating time (8–12 s), which is consistent with the volatilization time of PDBC (Figs. S5 and S6). In comparison, the solvent additive provides a stable aggregation process, which is reflected by the PM6_0-0_ absorption peak position that hardly changes after 8 s. However, the absorption intensities in additive-treated PM6 films are mostly lower than the control, which contributes to a low *J*_SC_ in the devices that discusses later. This is another motivation for us to blend the additives with the acceptor material in the upper solution in SqP.

For PYF-T-*o* film, all samples show similar redshifts and reduced absorption intensities of the PYF-T-*o*_0-0_ peak within ~ 5 s of spin-coating, indicating that the crystallization/aggregation of PYF-T-*o* phase begins in the initial solvent evaporation stage. Compared with pure PYF-T-*o*, the drying time is prolonged after using ODBC or PDBC, but the redshift or blueshift on the PYF-T-*o*_0-0_ peak in final solid film is not remarkable. The result suggests that the* E*_PYF-T-*o*:additive_ may be too small to adjust the PYF-T-*o* aggregation behavior. Among them, the PYF-T-*o*_0-0_ peak position in ODBC-treated sample exhibits a smooth redshift over 0–4 s, which can be regarded as a signal of homogeneous nucleation. Similarly, the absorption intensities of the additives-treated PYF-T-*o* films are slightly reduced compared to the control, which is consistent with the result of the PM6:additive films. The PM6 or PYF-T-*o* film processed with the liquid additive (ODBC) presents a smaller sacrifice in absorbance compared with the solid additive (PDBC), which should increase the *J*_SC_. Furthermore, we investigate the surface morphology of film using atomic force microscopy (AFM). The height and phase images of PM6:additives and PYF-T-*o*:additives films are shown in Fig. S7. For films processed with the ODBC additive, the surface root mean square (*R*q) of PM6 and PYF-T-*o* films are 1.91 and 1.44 nm, respectively, which are significantly lower than those without (2.42 and 1.65 nm) or with the solid additive, PDBC (4.54 and 2.63 nm).

**Fig. 2:**
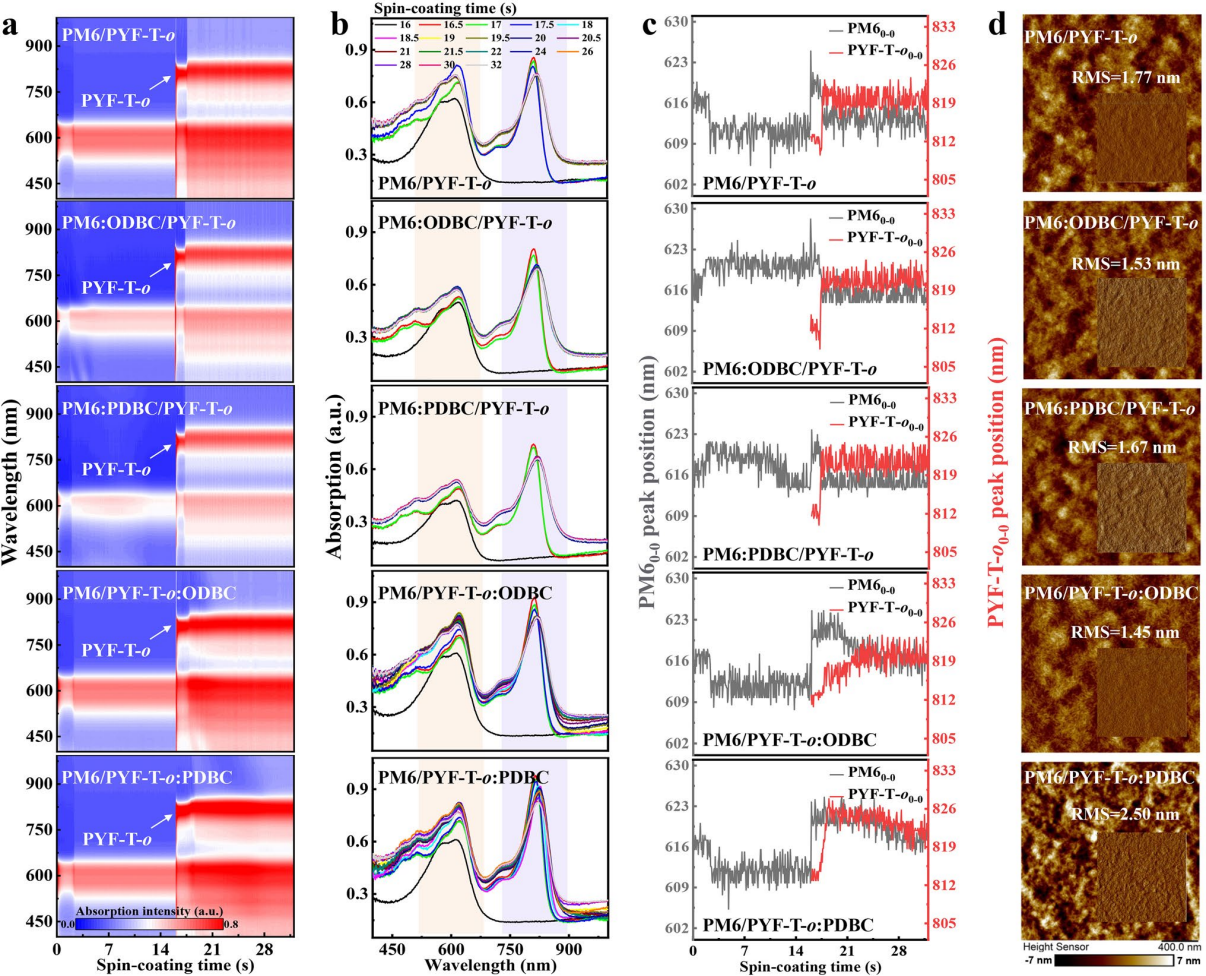
**a** Absorption intensity change over time monitored by in-situ absorption spectra of the SqP PM6/PYF-T-*o* films during spin-coating, where the ODBC or PDBC additives are added either into the PM6 or PYF-T-*o* precursor solution. **b** Absorption spectra at representative times during or after spin-coating. **c** Evolution of PM6_0-0_ and PYF-T-*o*_0-0_ peak positions in PM6/PYF-T-*o* films. **d** AFM height images of the SqP PM6/PYF-T-*o* films with the corresponding phase images inserted

Subsequently, *in-situ* absorption spectra of PYF-T-*o* solution spin-coated onto the PM6 underlayer were measured for revealing the effect of additives on the interdiffusion between donor and acceptor in SqP (Figs. 2a-c and S8). It can be seen that the solid film of PM6:additive formed within 16 s, followed by the PYF-T-*o* solution being deposited (> 16 s) on top, when the PM6 underlayer entered back into the liquid phase demonstrated by the PM6 absorption peak (620 nm) decreasing with time. This can be viewed as that the PM6 layer was entirely swelled by the acceptor solution and got backed into solid state again upon drying with acceptor molecules infiltrated inside, which we call a *secondary nucleation* process for PM6. No matter whether additive was added to PM6 when preparing the underlayer, the times of secondary nucleation for PM6 are almost identical, i.e., the PM6:additives/PYF-T-*o* films shows similar secondary nucleation dynamics. This means that the *secondary nucleation* is not greatly influenced by the aggregation behavior of the already-formed PM6 underlayer. In contrast, when ODBC or PDBC was added into the acceptor (PYF-T-*o*) solution, the *secondary nucleation* process of PM6 and the drying time for the acceptor are simultaneously prolonged. This is extremely important for the SqP devices: In the SqP method, the introduction of additives into the upper acceptor solution can regulate the film formation kinetics of donors and acceptors concurrently. In particular, the PM6/PYF-T-*o*:ODBC film exhibits a smallest RMS of 1.45 nm (Fig. 2d), which is consistent with the results of Fig. S7. To explore the effect of isomeric additives on molecular stacking and orientation, Grazing incident wide-angle X-ray scattering (GIWAXS) experiments are conducted. The 2D GIWAXS patterns and the corresponding line-cut profiles in the in-plane and out-of-plane directions are shown in Fig. S9, with detailed peak and fitting data shown in Table S2. Compared with other four films, strong *π*–*π* stacking (010) peak in the *q*_z_ direction at ~ 1.712 Å^−1^ can be found in PM6/PYF-T-*o*:ODBC. Furthermore, the *d*-spacing in the *π*–*π* stacking direction (*d*_π_) and crystal coherence length (CCL) of three active layer films at the *q*_z_ direction are also extracted based *d*_π_ = 2*π*/*q*_z_ and CCL = 2*πK*/FWHM, respectively. The pristine PM6/PYF-T-*o* film and PDBC-treated sample film show a *d*_π_ of ~ 3.74 Å and a CCL of 9.64–10.04 Å. Notably, the PM6:ODBC/PYF-T-*o* and PM6/PYF-T-*o*:ODBC film possess the smaller *d*_π_ of ~ 3.67 Å and the longer CCL of ~ 11.14 Å, indicating that the ODBC additive can induce extended yet tight π-π stacking.

### Device Performance Analysis

**Fig. 3:**
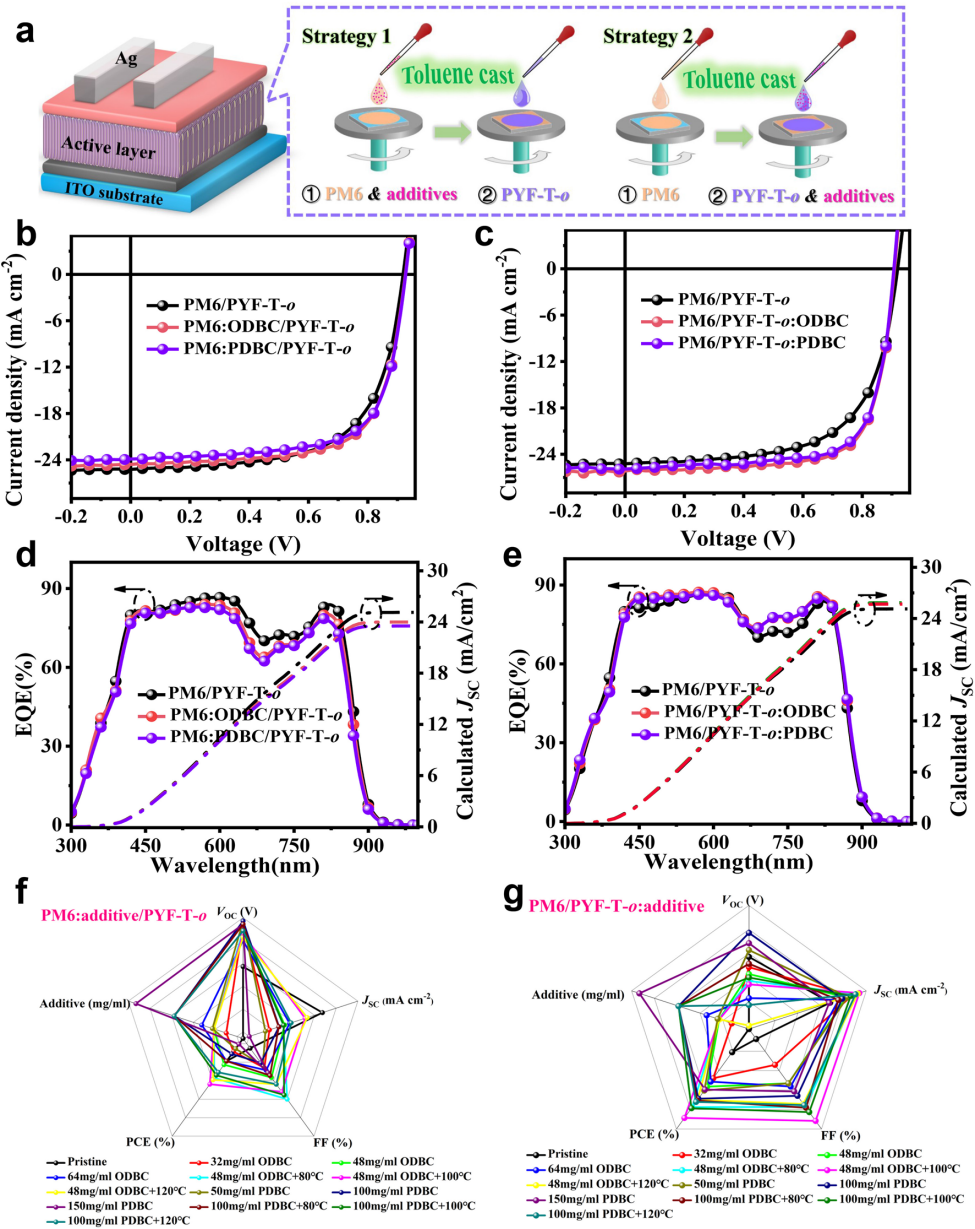
**a** Schematic diagram of SqP OSCs whose donor and acceptor are both processed from toluene with or without additive. **b**, **c** *J*–*V* characteristics and **d**, **e** EQE spectra of PM6:additive/PYF-T-*o* and PM6/PYF-T-*o*:additive SqP devices. Radar charts of photovoltaic parameters for **f** PM6:additive/PYF-T-*o* and **g** PM6/PYF-T-*o*:additive devices processed from different conditions. Absorption intensity change over time monitored by in-situ absorption spectra of the SqP PM6/PYF-T-*o* films during spin-coating, where the ODBC or PDBC additives are added either into the PM6 or PYF-T-*o* precursor solution

SqP devices with an ITO/PEDOT:PSS/PM6/PYF-T-*o*/PNDIT-F3N/Ag structure were fabricated to study the impact of the two isomeric additives on device performance. Figure 3a is the schematic diagram of OSCs preparation by combing the SqP method and non-halogen solvent, highlighting where the additive was added in. The detailed device optimization process can be shown in Figs. S10-S17 and Tables S3-S8 with radar charts in Fig. 3f, g summarizing these optimizations. The current density–voltage (*J*–*V*) curves and external quantum efficiency (EQE) spectra of the representative devices are show in Figs. 3b, d and c, e, respectively. For pristine PM6/PYF-T-*o* devices, a *J*_SC_ of 25.26 mA cm^*−*2^, an FF of 63.95% and a PCE of 14.88% are obtained (Fig. 3b and Table 1). When isomeric additive was added into the PM6 layer, we observe a large decrease in *J*_SC_: The SqP devices ODBC and PDBC added into the PM6 underlayer show *J*_SC_s of 24.70 and 24.01 mA cm^*−*2^, respectively, which is mainly resulted from their lower absorption coefficients (Fig. 2a, b). Meanwhile, the photo-responses ranging from 450 to 620 nm in EQE spectra for devices with additives added in the donor, i.e., PM6:ODBC/PYF-T-*o* and PM6:PDBC/PYF-T-*o*, are lower than those of the control (no additive), which is highly consistent with the absorption spectra in Fig. 1c, d where we found reduction in optical density caused by the insertion of the additives in the donor. The PCEs for SqP devices with ODBC and PDBC in the PM6 layer are similar (15.78% for ODBC and 15.43% for PDBC), both are higher than the control device (14.88%) mainly due to the enhanced FF. Figure 3c shows that the *J*_SC_ and FF for devices with additives added into the acceptor solution (PM6/PYF-T-*o*:additive) are concurrently promoted due to the strong light absorption and improved morphology that will be discussed in later sections. In particular, the PM6/PYF-T-*o*:ODBC device shows a higher *J*_SC_ of 26.18 mA cm^*−*2^ and a higher FF of 72.95% than those of the PDBC-treated devices (25.91 mA cm^*−*2^ and 72.14%) and commercial 1-chloronaphthalene devices (25.49 mA cm^−2^ and 72.47%), indicating that ODBC can serve as an alternatives for traditional 1-chloronaphthalene. The high *J*_SC_ value is mainly related to the enhanced photoresponse in the range of 700–900 nm (Fig. 3e). Small differences (< 3%) between the EQE-integrated and *J*–*V* measured *J*_SC_ values are obtained (Table 1). Thus, a high PCE of 17.38% is obtained for the toluene-processed binary all-polymer solar cell based on the PM6/PYF-T-*o*:ODBC active layer, which also surpasses the PM6:PYF-T-*o* devices prepared using traditional blend-casting method from chloroform solvent (16.81%). Meanwhile, the PCE value of 17.38% is also the highest efficiency so far in binary PM6:PYF-T-*o* system. In addition, we also evaluated devices with additives introduced into both solutions: The optimal ODBC additives added into both PM6 and PYF-T-*o* layers simultaneously demonstrates a PCE ranging from 12.54% to 16.53% (Fig. S17 and Table S8). This variation in performance can be attributed to the excessive phase separation induced by ODBC, which leads to significant reductions in both *J*_SC_ and FF [36, 37]. For blend-casting method, we also try the potential of ODBC and PDBC additives in toluene-processed device (Fig. S18 and Table S9). Although the efficiency of blended devices processed with ODBC or PDBC is less than SqP-based devices, it nonetheless remains substantially higher PCE values (15.33%–15.78%) than that of PM6:PYF-T-*o* without additives (12.81%).

**Table 1:** Summary of photovoltaic device parameters

Active layer	*V*_OC_ (V)	*J*_SC_ (mA cm^−2^)	FF (%)	PCE (%)
PM6/PYF-T-*o*	0.921	25.26 (25.12)^a^	63.95	14.88^b^ (14.61^c^ ± 0.11)
PM6:ODBC/PYF-T-*o*	0.926	24.70 (24.00)^a^	69.01	15.78^b^ (15.72^c^ ± 0.08)
PM6:PDBC/PYF-T-*o*	0.927	24.01 (23.53)^a^	69.37	15.43^b^ (15.26^c^ ± 0.10)
PM6/PYF-T-*o*:ODBC	0.910	26.18 (25.85)^a^	72.95	17.38^b^ (17.21^c^ ± 0.07)
PM6/PYF-T-*o*:PDBC	0.909	25.91 (25.69)^a^	72.25	17.01^b^ (16.83^c^ ± 0.19)

^a^Values in brackets are integrated from EQE spectra

^b^Based on the devices with maximum PCE

^c^The averages are extracted from at least 5 independent devices

**Fig. 4:**
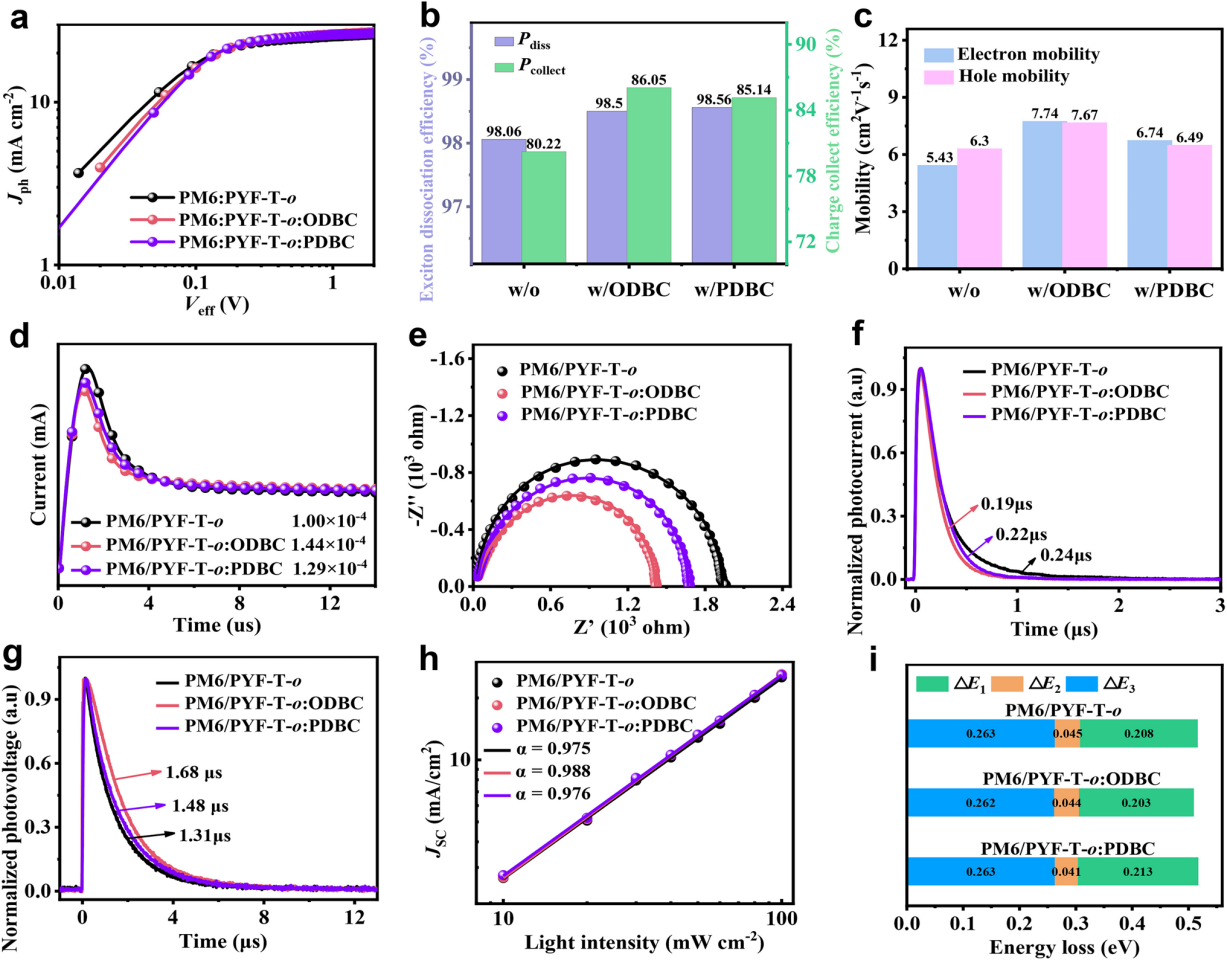
**a** *J*_ph_–*V*_eff_ curves. **b** Extracted *P*_diss_ and *P*_collect_ parameters. **c** Summary of electron and hole mobility from single carrier devices. **d** Photo*-*CELIV results. **e** Impedance spectroscopy results. **f** TPC, **g** TPV, and **h** *J*_SC_–*P*_light_ curves and **i** *E*_loss_ parameters of three PM6/PYF-T-*o* devices processed by the SqP method

It can be clearly seen that the photovoltaic parameters of SqP device can reach a better level when additives are added into the casting process of the acceptor layer. Afterwards, we selectively analyzed the difference between the PM6/PYF-T-*o* and PM6/PYF-T-*o*:additive devices in carrier dynamics. To understand the effect of morphology on device physics, we conducted a series of optoelectronic measurements. First, the photocurrent density*-*effective voltage (*J*_ph_–*V*_eff_) curves of PM6/PYF-T-*o*:additive devices are shown in Fig. 4a. *J*_ph_ and *V*_eff_ are defined by the *J*_ph_ = *J*_light_* − J*_dark_ and* V*_eff_ = *V*_applied_* − V*_0_ equations, respectively. Here, *J*_light_ and *J*_dark_ are the current density of device under illumination and dark conditions, respectively. *V*_applied_ and *V*_0_ refer to the applied voltage across the device and the voltage under which *J*_ph_ equals zero [38]. At *V*_eff_ = 1.5 V, *J*_ph_ in PM6/PYF-T-*o* devices treated by isomeric additives reaches a saturation value (*J*_sat_), which can be used to calculate exciton dissociation rate (*P*_diss_ = *J*_ph_/*J*_sat_) and charge collection efficiency (*P*_collect_ = *J*_max_/*J*_sat_) under the short-circuit and maximum power output conditions (Fig. 4b) [39]. The *P*_diss_ extracted from *J*_ph_–*V*_eff_ curves are 98.06%, 98.5%, and 98.56%, for PM6/PYF-T-*o*, PM6/PYF-T-*o*:ODBC, and PM6/PYF-T-*o*:PDBC respectively, and the corresponding* P*_collect_s are 80.22%, 86.05%, and 85.14%. Meanwhile, the maximum exciton generation rates of PM6/PYF-T-*o*:additive device are also calculated based on the empirical formula, *G*_max_ = *J*_sat_/*eL* (Table 2), where the for *G*_max_ for the devices without additive, with ODBC in acceptor and with PDBC in acceptor are 1.61 × 10^28^, 1.66 × 10^28^, and 1.64 × 10^28^ m^−3^ s^−1^, respectively. The ODBC-treated OSCs achieve the largest *P*_collect_ and the highest* G*_max_, signifying the least amount of carrier recombination.

**Table 2:** Summary of exciton dissociation, charge carrier transport and recombination parameters

Additive	*P*_diss_^a^ (%)	*P*_collect_^a^ (%)	*G*_m_^a^ (10^28^ m^−3^ s^−1^)	*μ*_e_/*μ*_h_^b^ (10^–4^ cm^2^ V^−1^ S^−1^)	*μ*_avg_^c^ (10^–4^ cm^2^ V^−1^ S^−1^)	Ratio^d^	*τ*_extract_^e^ (μs)	*τ*_lifetime_^f^ (μs)	*α*^g^
Without	98.06	80.22	1.61	5.43/6.3	1.00	0.86	0.24	1.31	0.975
ODBC	98.50	86.05	1.66	7.74/7.67	1.44	1.01	0.19	1.68	0.988
PDBC	98.56	85.14	1.64	6.74/6.49	1.29	1.04	0.22	1.48	0.976

^a^Calculated based on *J*_ph _–*V*_eff_ curves

^b^Calculated based on *J *− *V* curves of electron-only devices and hole-only devices

^c^Extracted from Photo-CELIV curves

^d^The ratio is obtained from *μ*_e_/*μ*_h_

^e^Obtained from fitting TPC curves

^f^Acquired from TPV curves

^g^Derived from the JSC-Plight curves fitting analysis

To evaluate the charge transport properties, we first fabricated electron-only and hole-only devices and employed the space charge limited current (SCLC) model to estimate the charge carrier mobilities (Fig. S19). The electron-only and hole-only devices are fabricated with structures of ITO/ZnO/PM6/PYF-T-*o*:additive/PNDIT-F3N/Ag and ITO/PEDOT:PSS/PM6/ PYF-T-*o*:additive/MoO_3_/Ag, respectively. The mobility is obtained using Mott-Gurney model, $J=\frac{9{\varepsilon }_{0}{\varepsilon }_{r}\mu {V}^{2}}{8{d}^{3}}$ whose results are shown in Fig. 4c [40]. The electron mobility (*μ*_e_) and hole mobility (*μ*_h_) for the pristine PM6/PYF-T-*o* device are 5.43 × 10^–4^ and 6.3 × 10^–4^ cm^2^ V^−1^ S^−1^, respectively, which are the lowest among all conditions, suggesting that the miscibility of the donor and acceptor without additive is high and the crystalline domains may not be sufficient to form a continuous network. For additive-treated devices, the *μ*_e_ and *μ*_h_ are simultaneously enhanced owing to their optimized morphology and improved crystallinity/aggregation. Among them, the highest charge mobility is found in the PM6/PYF-T-*o*:ODBC devices, whose *μ*_e_ and *μ*_h_ are 7.74 × 10^–4^ and 7.67 × 10^–4^ cm^2^ V^−1^ S^−1^, respectively, higher than PM6/PYF-T-*o*:PDBC (6.74 × 10^–4^ and 6.49 × 10^–4^ cm^2^ V^−1^ S^−1^). In addition, the PM6/PYF-T-*o*:ODBC device also shows a more balanced charge transport on the other hand, with the *μ*_e_ and *μ*_h_ being 7.74 × 10^–4^ and 7.67 × 10^–4^ cm^2^ V^−1^ S^−1^, respectively, which should contribute to its high FF [41]. Moreover, the average carrier mobility in working devices is further elucidated using photo-charge carrier extraction by linear increasing voltage (photo-CELIV) measurement (Fig. 4d) [42, 43]. The average excess carrier mobility (*μ*_avg_) can be extracted from ${\mu }_{avg}={2d}^{2}/(3A{t}_{max}^{2}(1+0.36\Delta j/{j}_{0}))$, whose values are shown in Table 2, which are 1.00 × 10^–4^, 1.44 × 10^–4^, and 1.29 × 10^–4^ cm^2^ V^−1^ S^−1^ for PM6/PYF-T-*o*, PM6/PYF-T-*o*:ODBC, and PM6/PYF-T-*o*:PDBC devices, respectively, the trend of which being consistent with the SCLC analysis.

Next, the impedance spectra of the PM6/PYF-T-*o*:additive devices are measured under dark (Fig. 4e). The experiment is conducted at *V*_OC_ with a 50 mV perturbation, and the frequency range is from 100 Hz to 1 MHz. In Fig. 4e, the symbol denotes experimental data, while the solid lines are fitted data by the equivalent circuit (Fig. S20 and Table S10). The similarity in the *R*_s_ values for the devices is explained by their similar device structure, while *R*_1_ and *R*_2_ exhibit a significant decrease, which is likely resulted from the more phase-separated morphology and improved interface contact after using ODBC or PDBC as the additive. In particular, the smallest *R*_1_ (1218 Ω) and *R*_2_ (157.5 Ω) are obtained in PM6/PYF-T-*o*:ODBC device, meaning that the charge transfer is more efficient. In addition, the influence of additives on transient photocurrent (TPC) and transient photovoltage (TPV) decays is also investigated (Fig. 4f, g). By using the single exponential equation to fit the TPC and TPV curves, the shortest *τ*_extract_ (0.19 μs) and longest *τ*_lifetime_ (1.68 μs) are found concurrently in PM6/PYF-T-*o*:ODBC device, indicating more efficient charge extraction and longer carrier lifetime in these devices [44]. In addition, charge recombination in OSCs is first estimated by the dependence of *J*_SC_ on light intensity (*P*_light_), which follows the relationship of ${J}_{SC}\propto {P}^{\alpha }$, as shown in Fig. 4h [45]. Here, *α* is used for evaluating the degree of bimolecular recombination. After adding ODBC in the acceptor, the *α* reaches 0.988 that is closest to 1 compared to the control without additive (0.975) and the PDBC devices (0.976) [46, 47]. Furthermore, the current-based deep-level transient spectroscopy (DLTS) measurement is employed to understand the trap state density (Fig. S21). The experiment is carried at a reverse bias voltage of − 3.0 V lasting from 0.1 to 10 μs after applying a voltage step to the devices. Among them, the PM6/PYF-T-*o*:ODBC device exhibits a slightly lower trap state volume density (*N*_t_) of 1.80 × 10^15^ cm^−3^ compare with the control condition (2.88 × 10^15^ cm^−3^) and PM6/PYF-T-*o*:PDBC device (2.22 × 10^15^ cm^−3^). The results demonstrate that the PM6/PYF-T-*o*:ODBC device is advantageous for achieving reduced energy losses and enhanced charge transport characteristics.

Afterwards, Fourier transform photocurrent spectroscopy external quantum efficiency (FTPS-EQE) and electroluminescence-EQE (EL-EQE) are employed for assess the influence of isomeric additives on energy loss (*E*_loss_) (Fig. S22). The detailed results are shown in Table S11 and Fig. 4i. The PM6/PYF-T-*o*:ODBC device exhibits a smaller *qV*_loss_ of 0.509 eV compared with the control (0.516 eV) and the PDBC device (0.517 eV). Interestingly, the Δ*E*_3_ that is related to non-radiative recombination losses is reduced from 0.208 eV (control) to 0.203 eV (ODBC), indicating that the enlarged domain and tighter π-π stacking in PM6/PYF-T-*o*:ODBC can decrease non-radiative recombination [48–50].

**Fig. 5:**
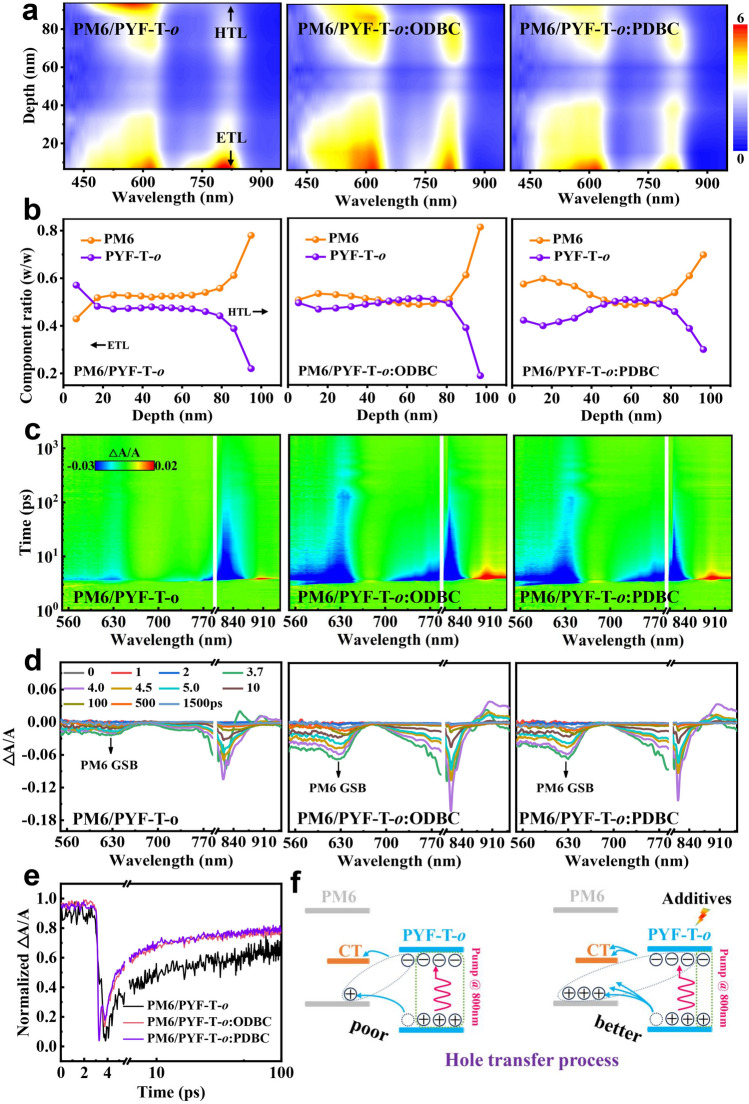
Devices studied are PM6:PYF-T-*o* with or without additive. **a** 2D contour maps of sublayer absorption spectra. The unit of scale bar is absorption intensity. **b** Compositional distribution of PM6 and PYF-T-*o* in the vertical direction of the active layer film. **c** 2D transient absorption (TA) spectra and **d** representative TA curves with an 800 nm excitation. **e** Normalized Δ*A*/*A* with respect to time monitored at 624 nm. **f** Schematic diagrams of the hole transfer process

To gain insight into the role of additives on the vertical phase segregation, we conducted film-depth-dependent light absorption spectroscopy (FDDLAS) measurements by monitoring the residual absorption during layer-by-layer plasma etching (Figs. S23 and S24) [51, 52]. The 2D contour maps of sublayer absorption spectra for the PM6/PYF-T-*o* and PM6/PYF-T-*o*:additive films are shown in Fig. 5a. For the control film, PM6/PYF-T-*o*, the photons absorbed by PM6 are concentrated in the top (~ 0–35 nm) and bottom (> 70 nm) regions, while the photons absorbed by PYF-T-*o* are mainly distributed at the bottom region (~ 0 to 30 nm). The nonuniform distribution could increase the difficulty of exciton dissociation. For the PM6/PYF-T-*o*:additive films, photons absorbed by donor and acceptor are more evenly distributed across the films, which is beneficial for exciton dissociation and charge transport. The most evenly distributed absorption profile in the regions of ~ 450 to 620 and ~ 750 to 850 nm are obtained in PM6/PYF-T-*o*:ODBC, whose *J*_SC_ is also the highest. Next, we calculated the exciton generation rates in the vertical direction for the active layer using a transfer matrix model (Figs. S25 and S26) [52, 53]. The exciton generation rates at the bottom and middle region is generally higher than the top region of the active layer, which is the result of the interplay between optical management and vertical phase segregation. Compared with the control without additive, the introduction of additive can improve exciton generation rates at the bottom region. At the depth of 50 nm, the PM6:PYF-T-*o*:ODBC film achieves a higher maximum exciton generation rate (*G*_max_) of 19.45 nm^−3^ s^−1^ than those of the control (18.10 nm^−3^ s^−1^) and PM6:PYF-T-*o*:PDBC film (18.32 nm^−3^ s^−1^). By integrating the exciton generation rate across the vertical direction, the highest overall exciton generation rate of 1.50 × 10^3^ nm^−2^ s^−1^ is obtained in the PM6:PYF-T-*o*:ODBC film, which contributes to its high *J*_SC_ as well.

Additionally, the distribution of the donor and acceptor material in the vertical direction can be revealed from the FLAS result. The composition ratio (Fig. 5b) of them is acquired by fitting the absorption profiles of the sublayers with the absorption spectra of neat donor and neat acceptor films (Fig. 1d). In the control film, the content of PM6 is slightly higher than PYF-T-*o* in the depth from 20 to 100 nm. For the PM6:PYF-T-*o*:ODBC film, the bottom (near anode) is extremely PM6-rich with a PM6 ratio reaching 81.16% compared with the control condition (78.02%), while the content of PYF-T-*o* near the top (cathode interface) is higher than PM6:PYF-T-*o*:PDBC (49.38% vs. 42.40%). This favorable vertical phase segregation is beneficial for achieving efficient charge transport and collection with reduced less interface recombination between the active film and the electrodes.

To more deeply reveal the impact of isomeric additives on the dissociation kinetics of the excitons, femtosecond transient absorption (TA) of the films is measured pumped at 800 nm. Two-dimensional (2D) TA spectra and representative TA curves are shown in Fig. 5c, d. A ground state bleaching signal (GSB) is observed at ~ 815 nm in the TA spectra, which matches well with the absorption of the PYF-T-*o* acceptor (Fig. 1d). Meanwhile, another negative GSB signal is found at 624 nm, which corresponds to the PM6 absorption, indicating the presence of a hole transfer process from PYF-T-*o* to PM6. The intensity of the GSB signal at 624 nm shows a significantly increase in PM6:PYF-T-*o*:additive films, corresponding to a larger amount of holes transferred from PYF-T-*o* to PM6 at the donor/acceptor interface. Furthermore, we also extracted TA decay kinetics of the films monitored at 624 nm to further quantify the differences in their hole transfer processes (Fig. 5e, f). The PM6:PYF-T-*o*:additive films show a higher hole transfer rate than the control sample. Compared with other sample films, the PM6/PYF-T-*o*:ODBC film simultaneously achieved the widest and highest GSB signal (624 and 815 nm), indicating ultrafast exciton dissociation and the generation of more free charges at the PM6/PYF-T-*o* interface, which is also consistent with its highest *J*_SC_ value.

### Device Stability and Universality

**Fig. 6:**
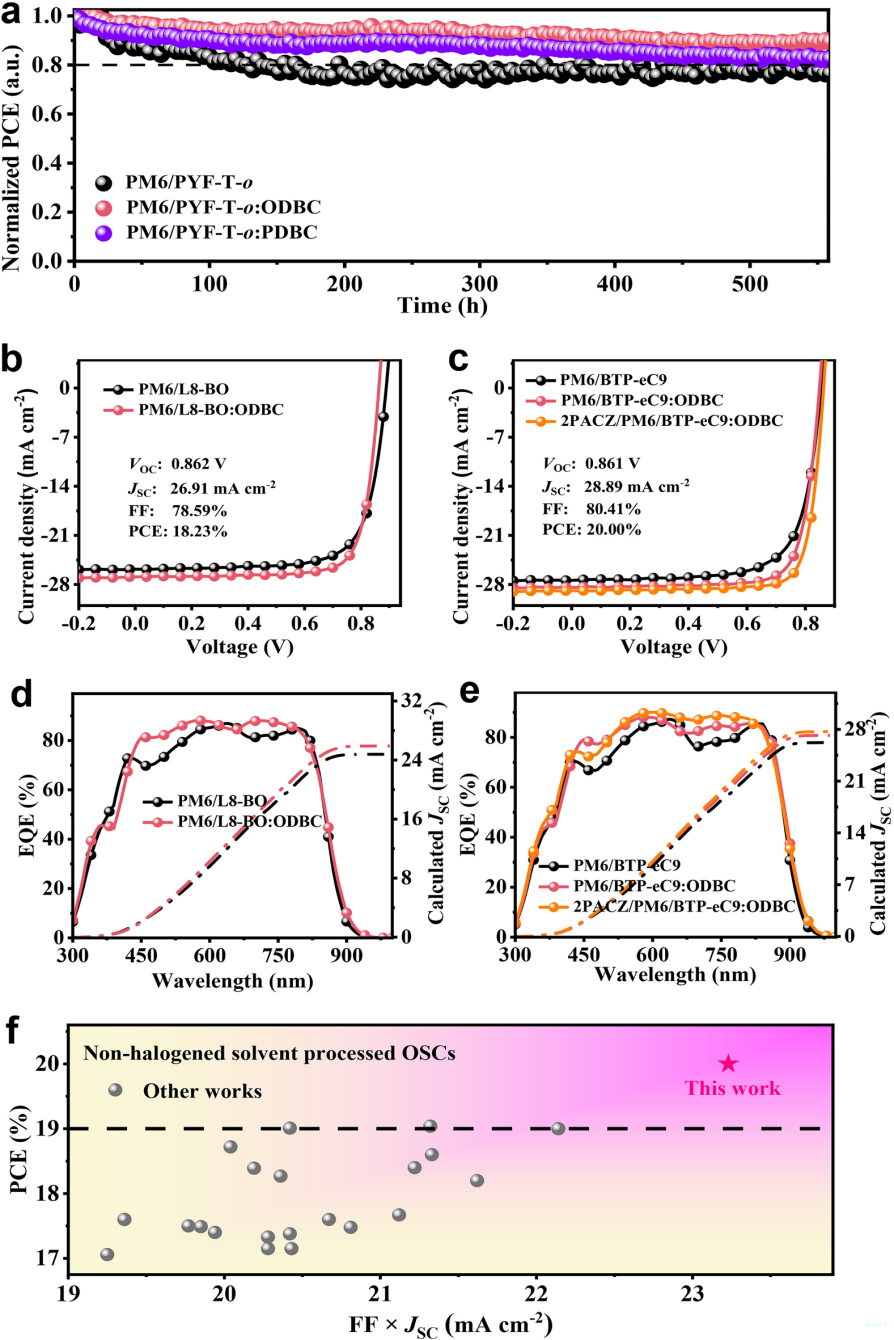
**a** Photostability of PM6/PYF-T-*o*:additives devices measured by MPPT method. **b**, **c** *J*–*V* characteristics and **d**, **e** EQE spectra of toluene-processed PM6/L8-BO and PM6/BTP-eC9 devices using either PEDOT:PSS or 2PACZ as the hole transport layer. **f** Comparison of PCE versus FF × *J*_SC_ for our result and literature result for non-halogen solvent processed binary OSCs

Then, we analyze the long-term light stability of the all-polymer solar cells by maximum power point tracking (MPPT) measurement under 1-Sun continuous LED illumination (Fig. 6a). For the control device, we noticed a rapid PCE decline with a poor *T*_80_ of 156 h. However, the decrease is remarkably reduced with the usage of additives: The PCE remains 88.4% (ODBC) and 82.6% (PDBC) of their initial values after 500 h of continuous light exposure. Moreover, thermal stability of the devices were tested (Fig. S27). The devices were placed on a heating stage at 80 °C. The PCEs of all devices show a sharp decay with heating time. After 75 h of heating, the PM6/PYF-T-*o*:ODBC device remains 59.35% of its initial PCE, which is slightly higher than those of the additive-free (51.26%) and PM6/PYF-T-*o*:PDBC device (51.31%). Overall, adding the asymmetric additive ODBC to the PYF-T-*o* solution increases both photo- and thermal stability of the device.

Finally, we evaluate the universality of the ODBC additive by using it in other binary OSCs including the PM6:L8-BO, PM6:BTP-eC9, and PM6/PJ1-γ devices (Figs. 6b-e, S28 and Table 3). Noted that we still adopt toluene as the main solvent for dissolving PM6, L8-BO, BTP-eC9, and PJ1-γ in the SqP method. Compared to the control (ODBC-free), adding ODBC in the acceptor solution can significantly promote the PCE. For instance, when PEDOT:PSS is used as the hole transport layer, the PCE of PM6/L8-BO and PM6/BTP-eC9 devices are increased to 18.16% and 18.81%, respectively, when ODBC is used in the upper acceptor layer. The optimization processes for high efficiency system based on PM6/BTP-eC9 can be shown in Fig. S29 and Table S12. For PDBC solid additive, we also observe an enhanced PCE of 17.60% and 18.41% for PM6/L8-BO and PM6/BTP-eC9 devices casted by toluene solvent (Fig. S30). In another all-polymer system utilizing PM6/PJ1-γ, the incorporation of the ODBC solvent additive demonstrates a superior PCE of 17.41% (Fig. S31 and Table S13). This performance surpasses that of the control device, which achieved a PCE of 14.36%, as well as the PM6/PJ1-γ:PDBC devices, which recorded a PCE of 16.93%. Notably, when the PEDOT:PSS layer is replaced by 2PACZ that tends to the self-assemble into a thin hole transport layer, the PCE of PM6/BTP-eC9 device is further increased to 20.00% (certified as 19.70% by the South China National Center of Metrology in China, Fig. S32), which is the highest efficiency of halogen-free solvent processed binary OSCs so far (Fig. 6f and Table S14). For large-area device (0.6 cm^2^), the PM6/BTP-eC9:ODBC device using PEDOT:PSS also obtained PCEs over 13% by combining SqP method and toluene solvent (Fig. S33).

**Table 3:** Photovoltaic parameters for PM6/L8-BO and PM6/BTP-eC9 devices using toluene as the main solvent

Active layer	*V*_OC_ (V)	*J*_SC_ (mA cm^−2^)	FF (%)	PCE (%)
PM6/L8-BO	0.888	25.82 (24.78)^a^	73.96	16.95^b^ (16.85^c^ ± 0.10)
PM6/L8-BO:ODBC	0.862	26.91 (25.90)^a^	78.59	18.16^b^ (18.05^c^ ± 0.07)
PM6/BTP-eC9	0.852	27.35 (26.22)^a^	72.26	16.84^b^ (16.82^c^ ± 0.04)
PM6/BTP-eC9:ODBC	0.847	28.41 (27.20)^a^	78.20	18.81^b^ (18.68^c^ ± 0.15)
PM6/BTP-eC9:ODBC^d^	0.861	28.89 (27.70)^a^	80.41	20.00^b^ (19.87^c^ ± 0.10)
PM6/BTP-eC9:ODBC^e^	0.861	28.91	79.20	19.70

^a^Integrated from EQE spectra

^b^Based on the device with the highest PCE

^c^Based on at least 5 independent devices

^d^With 2PACZ as the hole transport layer

^e^Certified by South China National Center of Metrology, China

## Conclusions

In summary, we introduce two highly volatile additives to finely tune the dynamic swelling-process of active layer based on non-halogen solvent and SqP method. It is found that additive can independently adjust the nucleation and drying kinetics of donor and acceptor through strong intermolecular non-covalent interaction, which further provides a guarantee for regulating the swelling process. It has been found that adding highly volatile additive to the later acceptor solution can simultaneously induce strong molecular aggregation and high absorption coefficients, solving the issue when additives are added into the donor. The PM6/PYF-T-*o*:additive film obtain an optimized π-π stacking, and improved vertical phase segregation. As a result of these morphological improvements, charge generation, charge collection, and charge extraction are greatly improved and carrier recombination significantly reduced. These merits lead to a champion PCE of 17.38% in the PM6/PYF-T-*o* all-polymer solar cell. Moreover, the method showed excellent generality in non-fullerene small molecule systems as well, including the L8-BO and BTP-eC9 systems. Also casted using toluene as the main solvent, and the PCE of PM6/BTP-eC9:ODBC device demonstrated an amazing PCE of 20.00% (certified 19.70%). Meanwhile, the photostability and thermal stability were both enhanced when ODBC was used. This work highly emphasizes the importance of non-covalent interactions in regulating the swelling process of SqP device. Introducing additives in the upper acceptor layer of sequential deposition is an effective method for achieving the high efficient non-halogen solvent processed OSCs.

## Supplementary Information

Below is the link to the electronic supplementary material.

Supplementary file1 (DOCX 21148 KB)

Supplementary file2 (MP4 10689 KB)
