# Supplementary material for: Achieving 20% Toluene-Processed Binary Organic Solar Cells via Secondary Regulation of Donor Aggregation in Sequential Processing
Source: Nanomicro Lett. 2025 Apr 1;17:206. doi: 10.1007/s40820-025-01715-2 (PMC11961838; doi:10.1007/s40820-025-01715-2)
Supplement: Supplementary file 1 — Supplementary file1 (DOCX 21148 KB) [file 40820_2025_1715_MOESM1_ESM.docx]

Supporting Information for

**Achieving 20% Toluene-Processed Binary Organic Solar Cells via Secondary Regulation of Donor Aggregation in Sequential Processing**

Yufei Wang^1, #^, Chuanlin Gao^1, #^, Wen Lei^2, #^, Tao Yang^3, #^, Zezhou Liang^4^, Kangbo Sun^1^, Chaoyue Zhao^1^, Lu Chen^1^, Liangxiang Zhu^1^, Haoxuan Zeng^1^, Xiaokang Sun^5^, Bin He^1^, Hanlin Hu^5^, Zeguo Tang^1^, Mingxia Qiu^1^, Shunpu Li^1^, Peigang Han^1^, and Guangye Zhang^1*^

^1^ College of New Materials and New Energies, Shenzhen Technology University, Shenzhen 518118, P. R. China

^2^ College of Cyber Security, Jinan University, Guangzhou 511443, P. R. China

^3^ Future Technology School, Shenzhen Technology University, Shenzhen 518118, P. R. China

^4^ Key Laboratory for Physical Electronics and Devices of the Ministry of Education & Shaanxi Key Lab of Photonic, Technique for Information, School· of Electronics Science & Engineering, Faculty of Electronic and· Information Engineering, Xi'an Jiaotong University, Xi'an 710049, P. R. China

^5^ Hoffmann Institute of Advanced Materials, Shenzhen Polytechnic University, Shenzhen 518055, P. R. China

^#^Yufei Wang, Chuanlin Gao, Wen Lei, and Tao Yang contributed equally to this work.

*Corresponding author. E-mail: [zhangguangye@sztu.edu.cn](mailto:zhangguangye@sztu.edu.cn) (Guangye Zhang)

**S1 Thermogravimetric analysis (TGA) and X-ray photoelectron spectra (XPS) measurements**

The TGA curves of ODBC solvent and PDBC solid additives are tested 40 to 200 ℃ at a scan rate of 10 ℃ min^−1^ (TGA/DSC3^+^, METTLER TOLEDO).The (XPS) The Br 3d Cl 2p signal the PM6/PYF-T-o:additive films before and after thermal annealing treatment were measured by XPS spectrometer (Escalab Xi^+^, Thermo Fisher Scientific (China) Co. Ltd)

**S2 Density Functional Theory (DFT) calculation**

The electrostatic potential (ESP) distribution and dipole moment of molecule are calculated based on the DFT under the B3LYP/6-31G(d,p) EM=GD3BJ level. For binding energy, the relevant DFT calculation was carried out using the B3LYP-(D3)BJ/def2-SVP level.

**S3 Device characterization**

The current density-voltage (*J*-*V*) curves of all encapsulated devices were measured using a Keithley 2400 Source Meter under AM 1.5G (100 mW cm^-2^) using an Enlitech solar simulator. The light intensity was calibrated using a standard Si diode with KG5 filter to bring spectral mismatch to unity. Optical microscope (Olympus BX51) was used to define the device area (3.95 mm²). EQEs were measured using an Enlitech QE-S EQE system equipped with a standard Si diode. Monochromatic light was generated from a Enlitech 300 W lamp source. Impedance spectroscopy and Photo-CELIV measurements were performed using commercially available Paios system (FLUXiM AG).

**S4 In situ UV-Vis absorption measurements**

In situ UV-Vis absorption measurements were carried out with a DU-100 system. The optical fiber was aligned so that the center of the light is focused on the center of the film. The sampling interval was 1 ms with an integration time of 5 ms per sample point. The average sampling times were adjusted between 4-7 times to obtain smooth absorption curves.

**S5 Atomic force microscopy (AFM)**

AFM measurements were performed by using a Scanning Probe Microscope Dimension 3100 in tapping mode under atmosphere conditions at room temperature. All film samples were spin-cast on glass/ITO substrates.

**S6 Grazing-incidence wide-angle X-ray scattering (GIWAXS) measurements**

GIWAXS were performed at beamline BL16B1 at the Shanghai Synchrotron Radiation Facility (SSRF). Samples were prepared on Si substrates using identical solutions as those used in devices. The 10k eV X-ray beam was incident for 30 s at a grazing angle of 0.15°, which maximized the scattering intensity from the samples. The scattered X-rays were detected using a Dectris Pilatus 1 M photon counting detector. 2D images were azimuthally averaged to obtain the reported one-dimensional (1D) scattering profiles using the Nika package. The coherence length was calculated using the Scherrer equation: CCL = 2πK/FWHM, where *K* is a dimensionless shape factor, normally with a value of 0.9. FWHM is the full with at half maximum of peak and *θ* is Bragg’s angle (scattering angle).

**S7 SCLC Measurements**

The electron-only devices with the structure of ITO/ZnO/PM6/PYF-T-*o*:additive/PNDIT-F3N/Ag and hole-only devices with the structure of ITO/PEDOT:PSS/PM6/PYF-T-*o*:additive/MoO_3_/Ag were prepared. The thickness of active layer is 100 nm. The *J*−*V* curves of electron-only and hole-only devices were measured in the dark condition with the analysis of SCLC model. The mobility is calculated based on the Mott-Gurney law ($J=\frac{9\varepsilon_{0}\varepsilon_{r}\mu V^{2}}{8d^{3}}$), where *J* is referred current density, *ε*_r_ stands for the relative dielectric constant in the organic photoactive layer (3.00), *ε*_0_ represents the vacuum permittivity of 8.85×10^−12^ F m^−1^, *d* is the organic layer thickness (100 nm), *V* is the applied voltage, and *μ* expresses mobility.

**S8 Transient photovoltage (TPV) and transient photocurrent (TPC) measurements**

In TPV measurements, the devices were placed under background light bias enabled by a focused Quartz Tungsten-Halogen Lamp with an intensity of similar to working devices, *i.e.,* the device voltage matches the open-circuit voltage under solar illumination conditions. Photo-excitations were generated with an 8 ns pulses from a laser system (Oriental Spectra, NLD520). The wavelength for the excitation was tuned to 518 nm with a spectral width of 3 nm. A digital oscilloscope was used to acquire the TPV signal at the open-circuit condition. TPC signals were measured under short-circuit conditions under the same excitation wavelength without background light bias.

**S9 Photo-charge carrier extraction by linear increasing voltage (Photo-CELIV) measurement**

According to the Juška et al. work [S1], a linearly increasing voltage (triangular pulse) is applied to extract thermally generated carriers and the mobility is obtained from the peak extraction time of the corresponding transient. However, the thermally generated carriers in organic semiconductors are low, thus, an input light pulse is typically employed to photogenerate the charges, when it is referred to as photo-CELIV. Under the excitation of light pulses, the real devices will produce electron and hole mobilities, and the CELIV peak is influenced by mobilities. Hence, the photo-CELIV peak provide an average mobility based on the equation, $\mu_{avg}={2d}^{2}/(3At_{max}^{2}(1+0.36\Delta j/j_{0}))$, where *d* is BHJ thickness, *A* voltage rise speed of the applied voltage pulse, *t*_max_ the time to reach the extraction current maximum, Δ*j* the displacement current, and *j_0_* initial current.

**S10 Deep-level transient spectroscopy (DLTS) measurement**

To gain information on trap state density, we carried out current-based deep-level transient spectroscopy (DLTS) measurement. Using the result of the measurement, the trap state density, *N*_t_, can be calculated based on the equation:

$$j_{te}\left( t \right)=2 \times\frac{1}{\tau_{te}}\times q\times d\times N_{t}\times exp(-\frac{t}{\tau_{te}})$$

where$j_{te}\left( t \right)$ is the trap emission current, $\tau_{te}$ the catch-trap emission time constant, *q* the elementary charge, *d* the film thickness, and *N_t_* the trap state density.

**S11 Film-depth-dependent light absorption spectroscopy (FLAS) and Femtosecond-resolved transient absorption (TA) spectra**

Film-depth-dependent light absorption spectra were acquired by an in-situ spectrometer (PU100, Shaanxi Puguang Weishi Co. Ltd.) (Shaanxi, China) equipped with a soft plasma-ion source. The power-supply for generating the soft ionic source was 100 W with an input oxygen pressure ~10 Pa. The film surface was incrementally etched by the soft ion source, without damage to the materials underneath the surface, which was in situ monitored by a spectrometer. From the evolution of the spectra and the Beer–Lambert’s Law, film-depth-dependent absorption spectra were extracted. Femtosecond-resolved TA spectra measurement was carried according to the reported literature [S2]. The intensity of the pump laser fluence is 0.7 µJ cm^−2^ at 800 nm excited light.

**S12 FTPS-EQE and EL-EQE measurements**

FTPS-EQE was measured using an integrated system (PECT-600, Enlitech), where the photocurrent was amplified and modulated by a lock-in instrument. EL-EQE measurements were performed by applying external voltage/current sources through the devices (REPS-Pro, Enlitech). All of the devices were prepared for EL-EQE measurements according to the optimal device fabrication conditions. EL-EQE measurements were carried out from 0 to 1.8 V.

**S13 Maximum power point tracking (MPPT) measurements**

The devices photostabilities were measured by using the solar cell stability test system (PR-SCCS, PURI Materials, China), and PURI2400-8Q as the source meters to record the solar-cells characteristics in an ambient atmosphere, which were conducted under continuous 100 mw cm^-2^ illumination provided by LED-solar simulators (PR-LEDSUN-8C, PURI Materials, China, with spectrum ranging from 350nm to 900nm). And the results were automatically recorded by the aging test software (PR-SCCS-MPPT, PURI Materials, China). The photostability experiment is conducted inside a glove box with N2, and the devices used for testing are not encapsulated.

**Supplementary Figures and Tables**


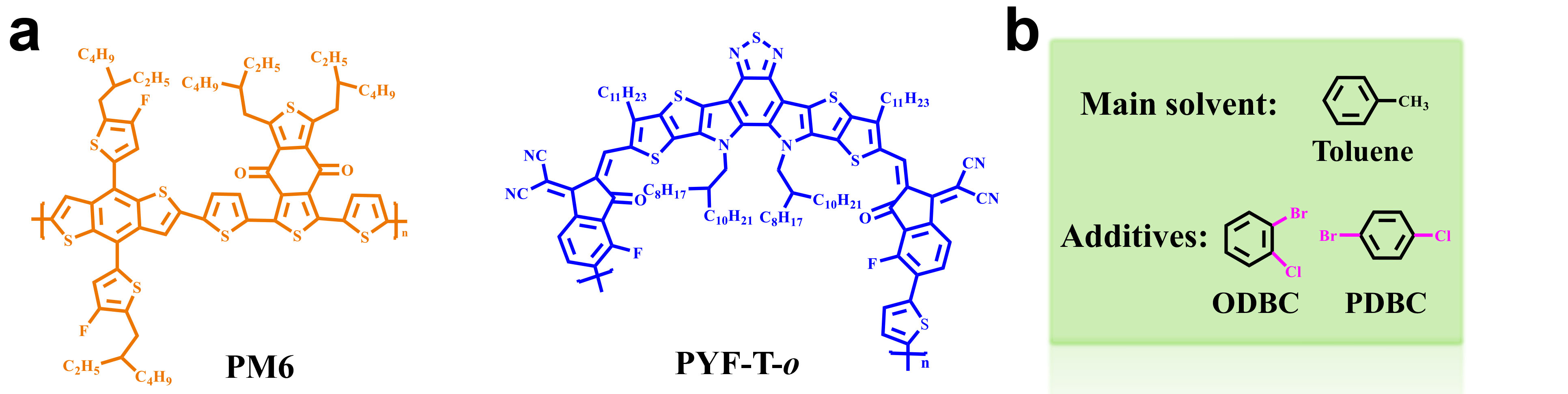


**Fig. S1** **a**) The chemical structures of all-polymer photoactive layers. **b**) The solvent and isomeric additive in this work

**Table S1** Physical properties of three isomeric additives

| Solvent | Boiling point [℃] | Melting point [℃] | Density [g ml^−1^] | State |
| --- | --- | --- | --- | --- |
| Toluene | 110.6 | -94.9 | 0.87 | solvent |
| ODBC | 204 | -13~-11 | 1.64 | solvent |
| PDBC | 196 | 64 | 1.65 | solid |


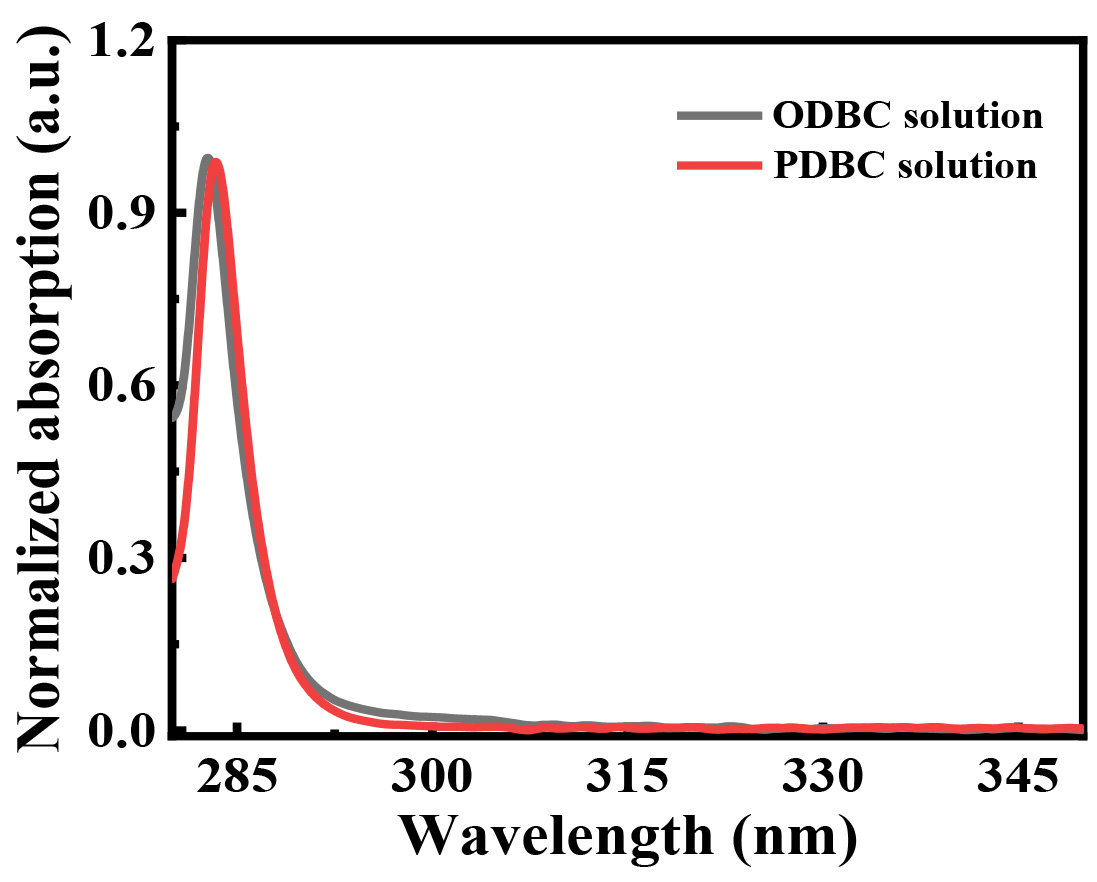


**Fig. S2** The normalized absorption spectra of ODBC and PDBC under solution condition


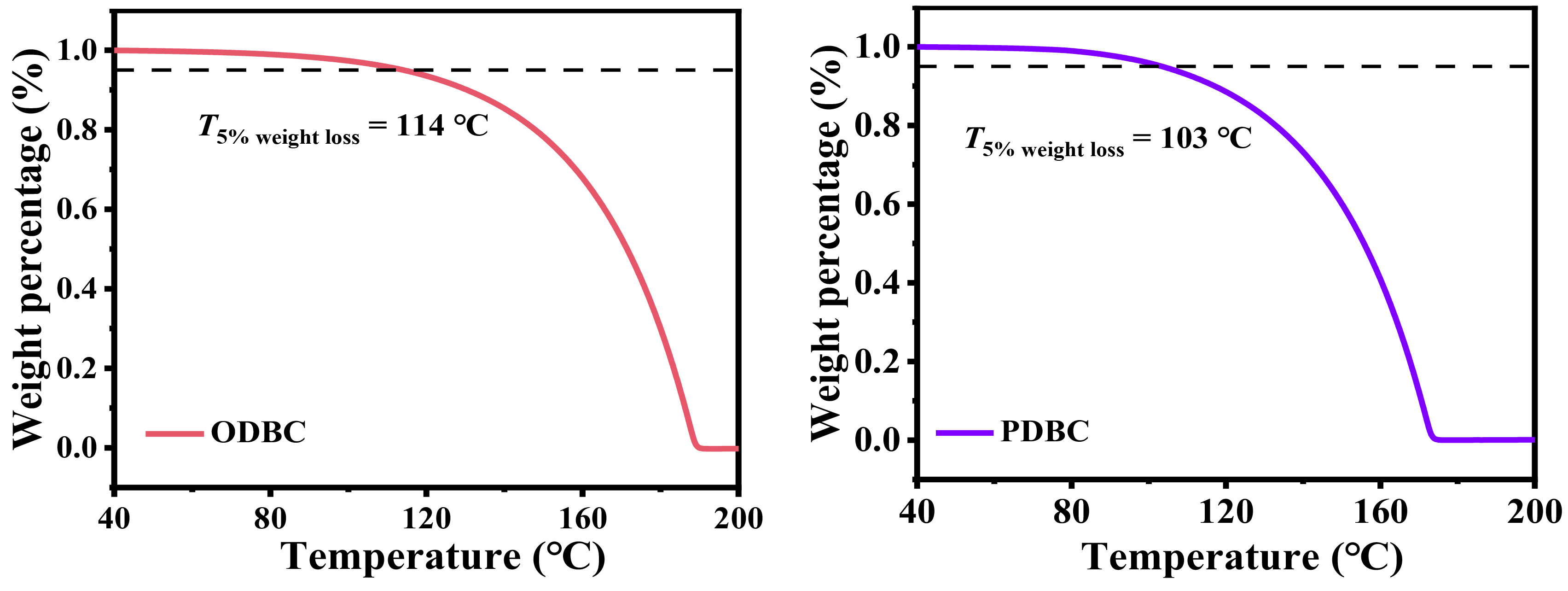


**Fig. S3** TGA curve of ODBC and PDBC heated from 40 to 200 ℃ at a scan rate of 10 ℃ min^−1^


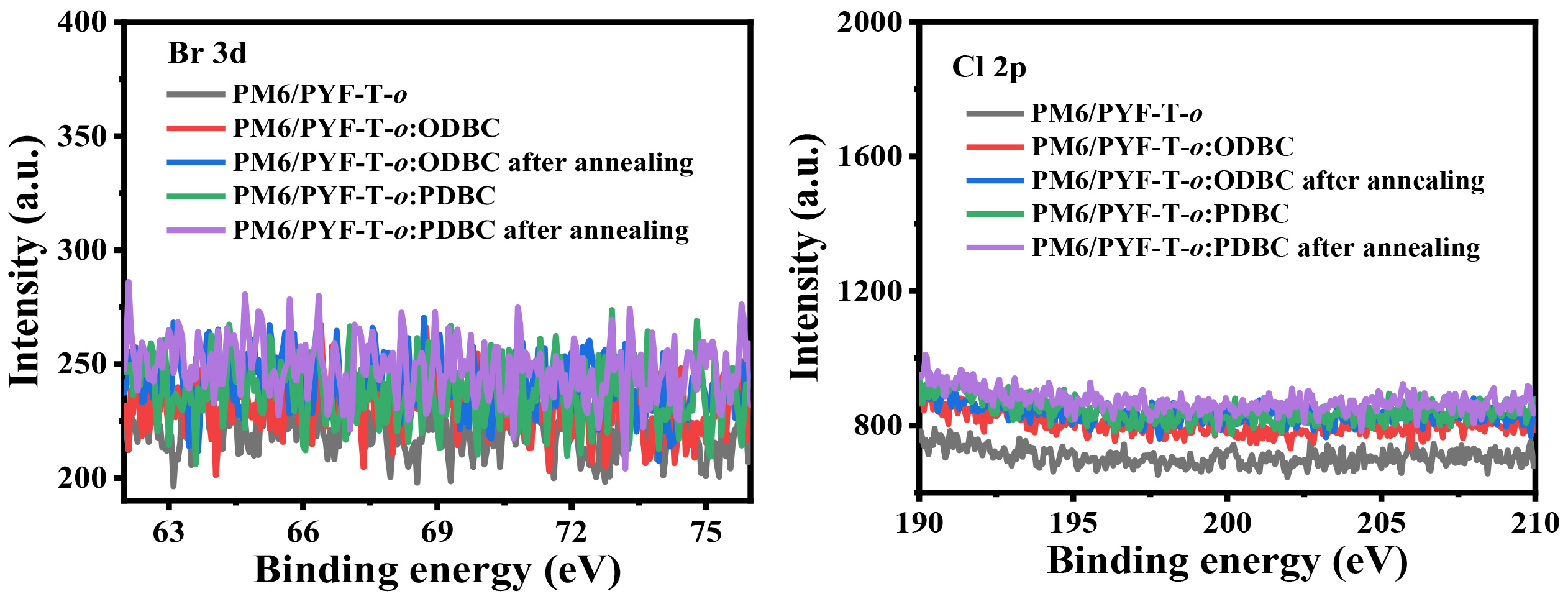


**Fig. S4** Left) Br 3d and right) Cl 2p XPS spectra of the PM6/PYF-T-*o*:additive films without or with thermal treatment at 100 ℃ for 5 min


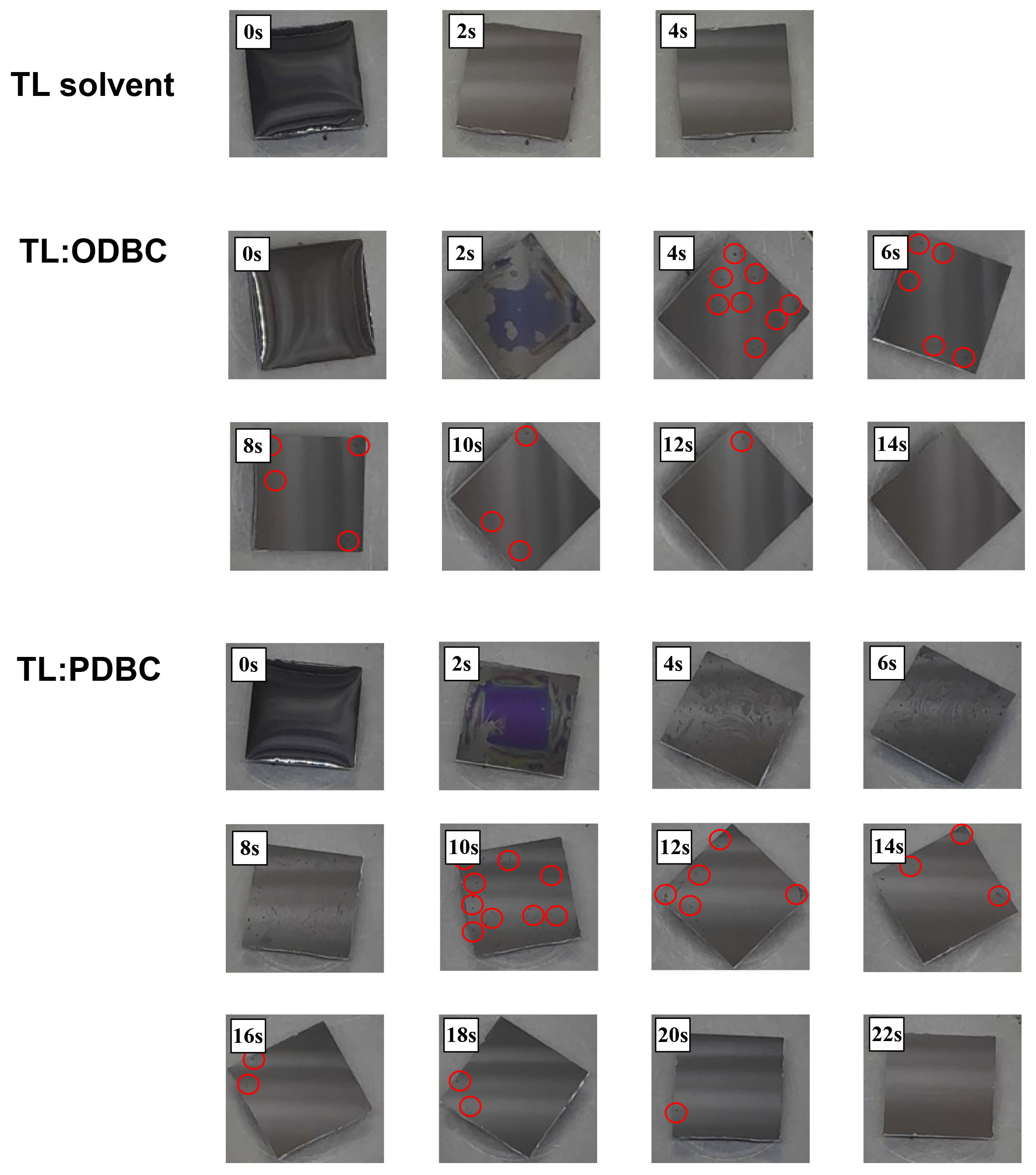


**Fig. S5** The photos of the toluene and toluene (TL): additive solution during spin-coating process. These photos are recorded every two seconds during spin-coating process


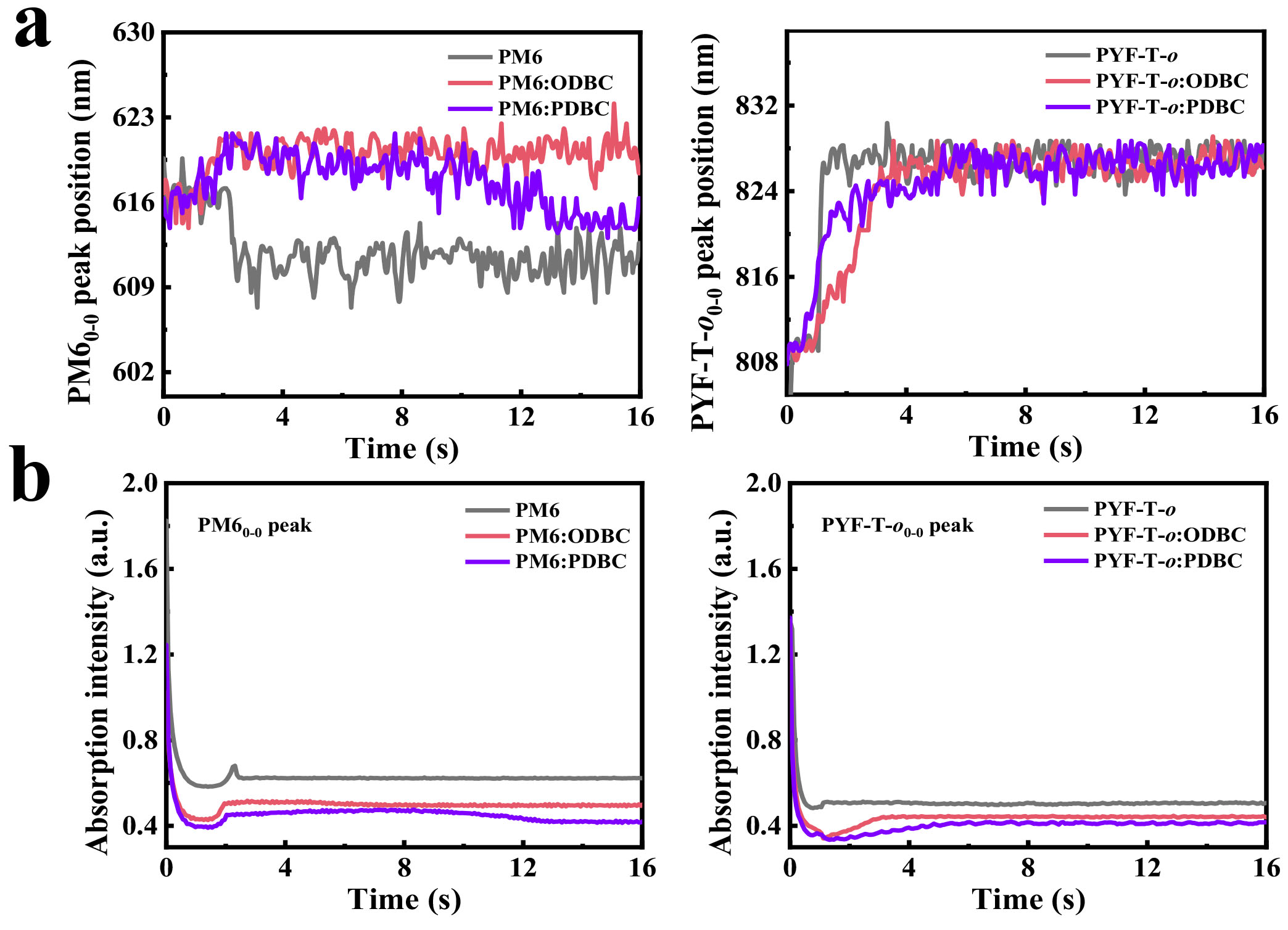


**Fig. S6** **a**) The PM6_0-0_ or PYF-T-*o*_0-0_ peak positions over spin-coating time. **b**) The absorption intensity of PM6_0-0_ or PYF-T-*o*_0-0_ peak over spin-coating time


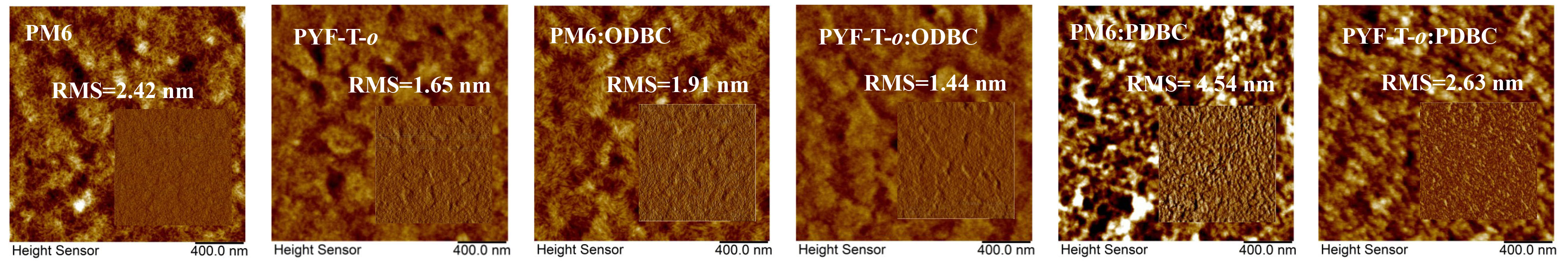


**Fig. S7** The height images and phase image of PM6:additive and PYF-T-*o*:additive films


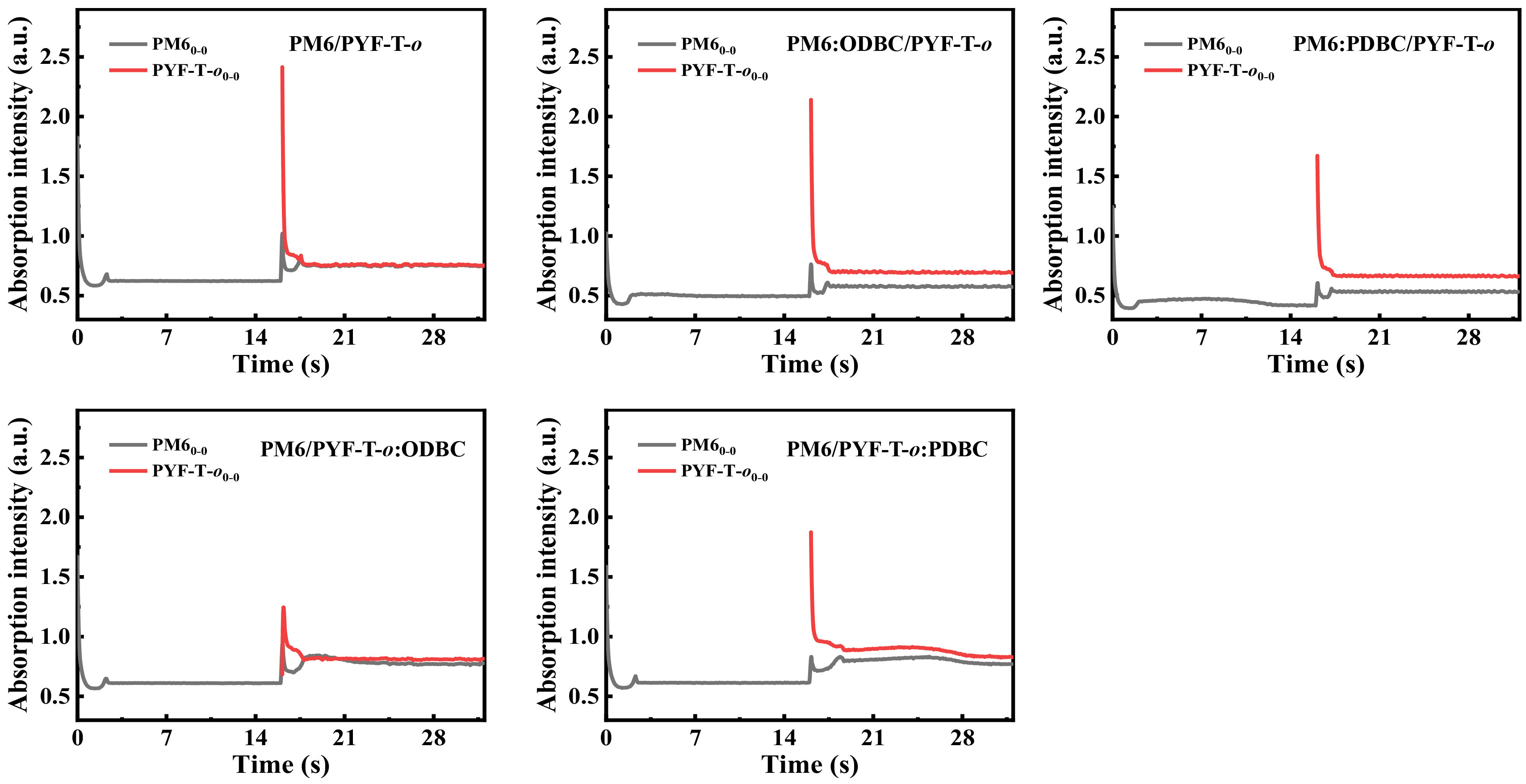


**Fig. S8** The absorption intensity of PM6_0-0_ or PYF-T-*o*_0-0_ peak in five sample films over spin-coating time


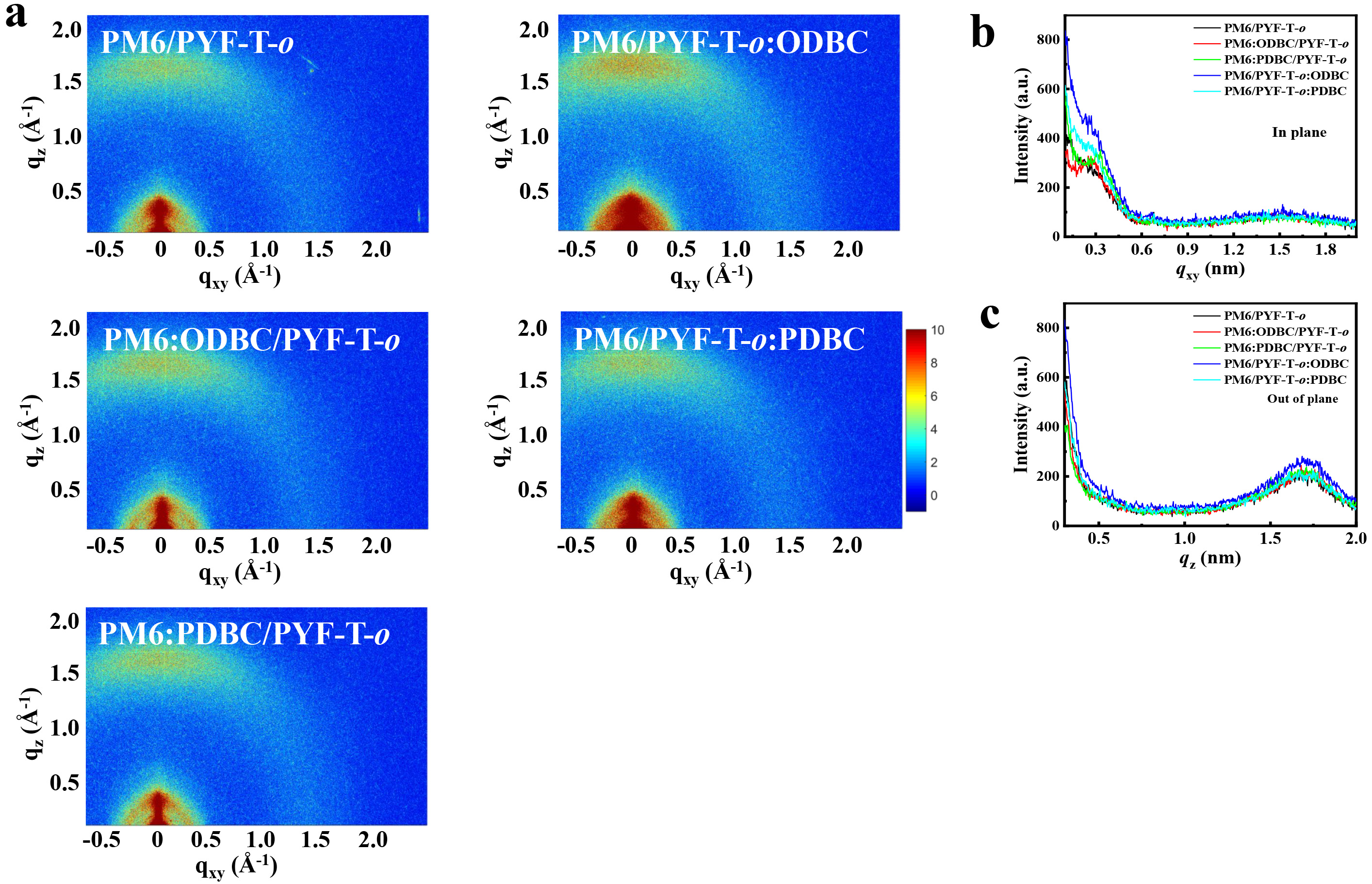


**Fig. S9** **a**) 2D GIWAXS pattern of PM6/PYF-T-o film without and with ODBC or PDBC treatment. The 2D scattering cut-line profiles of five PM6/PYF-T-*o* films: **b**) In-plane, **c**) out-of-plane

**Table S2** GIWAXS peak information of out-of-plane (010) of PM6/PYF-T-*o*:additive films

| Additive | Position [Å^-1^] | Intensity | *d*_π-π_ [A] | FWHM [Å^-1^] | CCL [Å] |
| --- | --- | --- | --- | --- | --- |
| PM6/PYF-T-*o* | 1.679 | 229 | 3.74 | 0.586 | 9.64 |
| PM6:ODBC/PYF-T-*o* | 1.706 | 255 | 3.68 | 0.501 | 11.28 |
| PM6:PDBC/PYF-T-*o* | 1.679 | 244 | 3.74 | 0.563 | 10.04 |
| PM6/PYF-T-*o*:ODBC | 1.712 | 291 | 3.67 | 0.507 | 11.14 |
| PM6/PYF-T-*o*:PDBC | 1.669 | 244 | 3.76 | 0.531 | 10.64 |


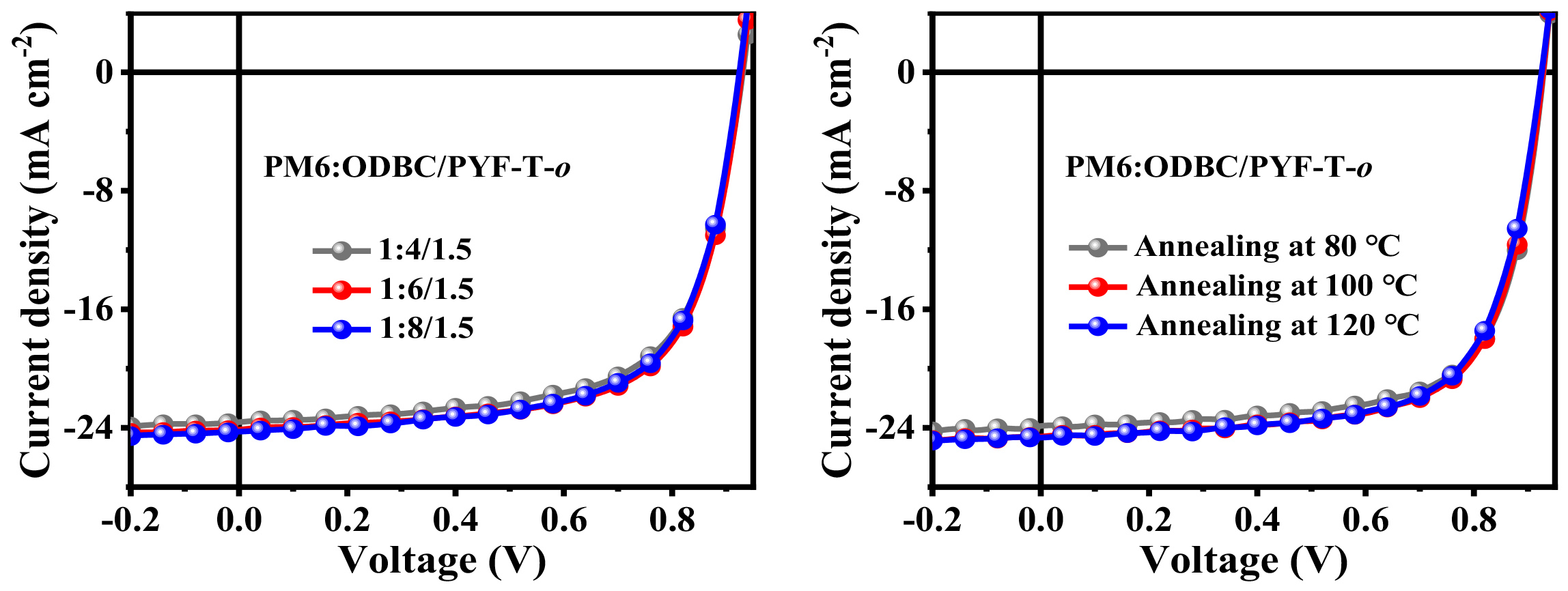


**Fig. S10** *J−V* curves of PM6:ODBC/PYF-T-*o* devices casted by SqP method without or with thermal annealing treatment

**Table S3** Photovoltaic parameters based on PM6:ODBC/PYF-T-*o* devices casted by SqP method with different treatment

| PM6:ODBC/PYF-T-*o* | Annealing | *V*_OC_ [V] | *J*_SC_ [mA cm^−2^] | FF [%] | PCE_max_ [%] |
| --- | --- | --- | --- | --- | --- |
| 1:4/1.5 | No | 0.929 | 23.55 | 66.31 | 14.51 |
| 1:6/1.5 | No | 0.927 | 24.04 | 67.40 | 15.02 |
| 1:8/1.5 | No | 0.924 | 24.20 | 66.58 | 14.88 |
| 1:6/1.5 | 80 ℃ | 0.927 | 24.00 | 69.84 | 15.54 |
|  | 100 ℃ | 0.926 | 24.70 | 69.01 | 15.78 |
|  | 120 ℃ | 0.925 | 24.77 | 68.17 | 15.61 |


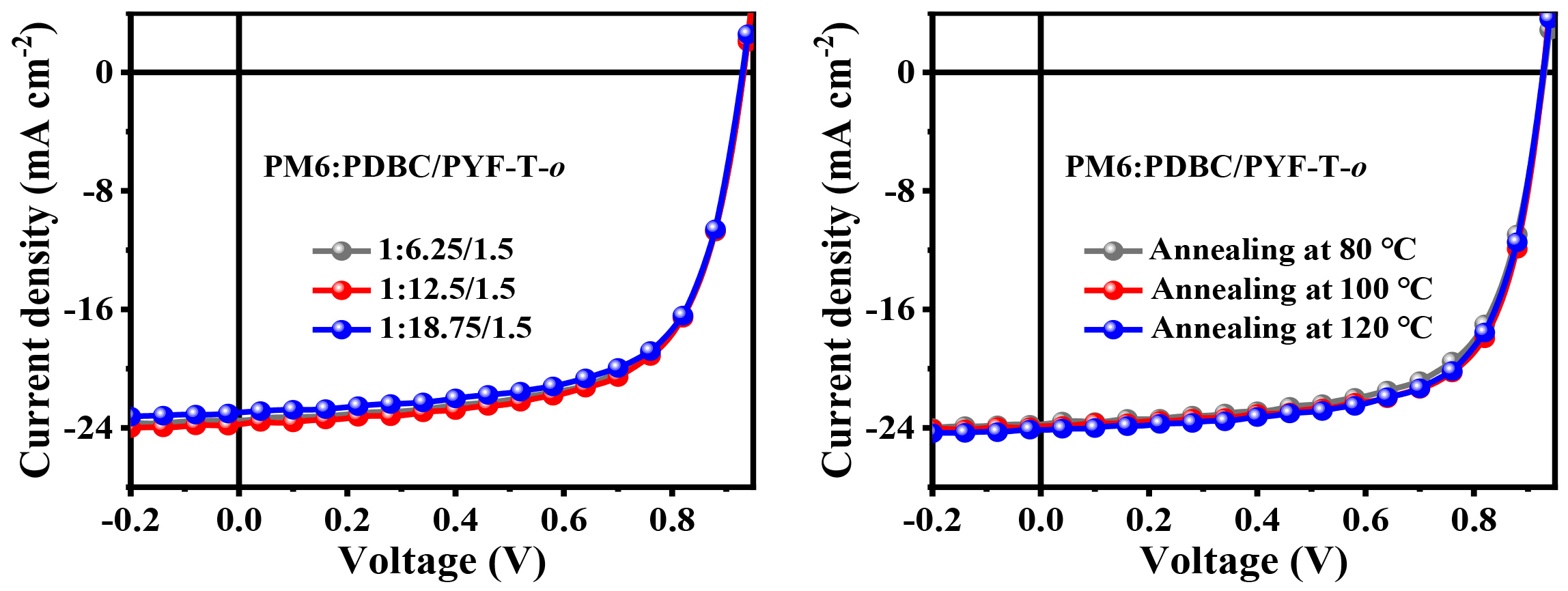


**Fig. S11** *J−V* curves of PM6:PDBC/PYF-T-*o* devices casted by SqP method without or with thermal annealing treatment

**Table S4** Photovoltaic parameters based on PM6:PDBC/PYF-T-*o* devices casted by SqP method with different treatment

| PM6:PDBC/PYF-T-*o* | Annealing | *V*_OC_ [V] | *J*_SC_ [mA cm^−2^] | | FF [%] | PCE_max_ [%] |
| --- | --- | --- | --- | --- | --- | --- |
| 1:6.25/1.5 | No | 0.930 | 23.42 | | 66.08 | 14.40 |
| 1:12.5/1.5 | No | 0.930 | 23.86 | | 65.75 | 14.60 |
| 1:18.75/1.5 | No | 0.929 | 22.90 | | 66.86 | 14.22 |
| 1:12.5/1.5 | 80 ℃ | 0.929 | 23.85 | | 67.18 | 14.88 |
|  | 100 ℃ | 0.927 | 24.01 | | 69.37 | 15.43 |
|  | 120 ℃ | 0.927 | | 24.25 | 68.15 | 15.32 |


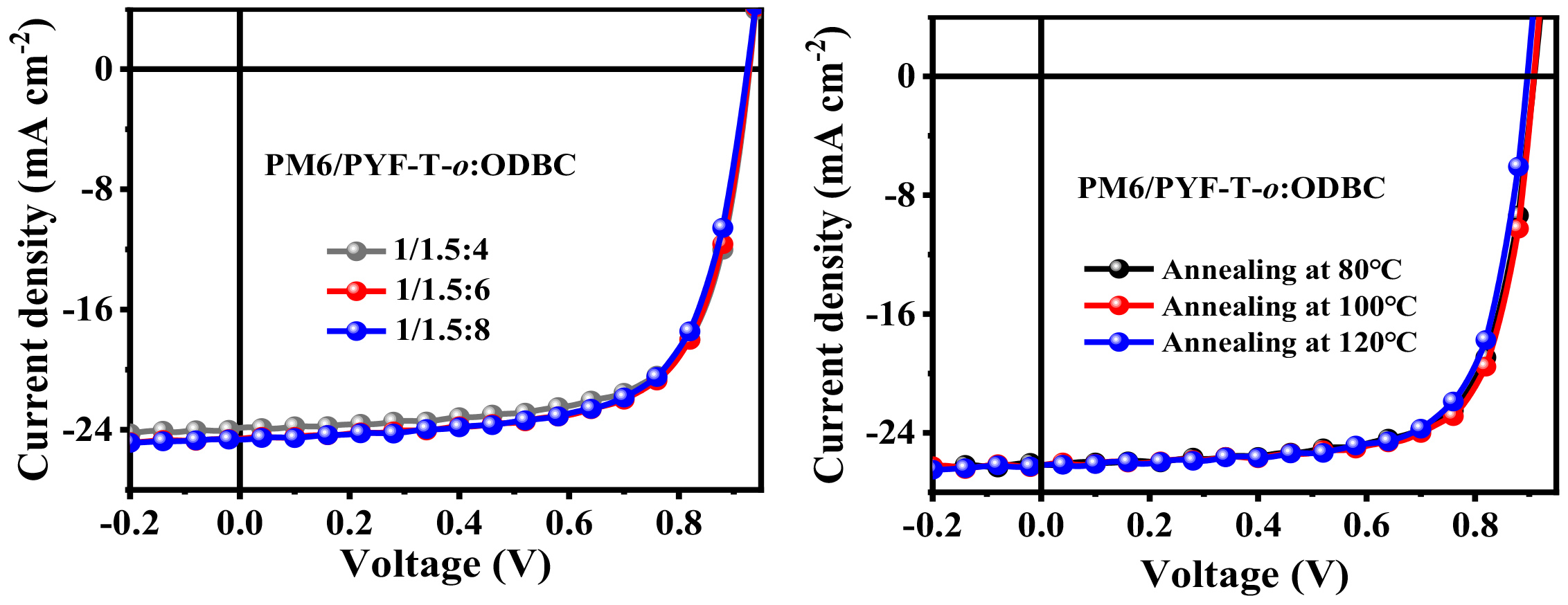


**Fig. S12** *J−V* curves of PM6/PYF-T-*o*:ODBC devices casted by SqP method without or with thermal annealing treatment

**Table S5** Photovoltaic parameters based on PM6/PYF-T-*o*:ODBC devices casted by SqP method with different treatment

| PM6/PYF-T-*o*:ODBC | Annealing | *V*_OC_ [V] | *J*_SC_ [mA cm^−2^] | FF [%] | PCE_max_ [%] |
| --- | --- | --- | --- | --- | --- |
| 1/1.5:4 | No | 0.913 | 25.95 | 66.94 | 15.87 |
| 1/1.5:6 | No | 0.911 | 25.77 | 68.97 | 16.19 |
| 1/1.5:8 | No | 0.904 | 25.53 | 69.32 | 16.00 |
| 1/1.5:6 | 80 ℃ | 0.909 | 26.09 | 71.57 | 16.97 |
|  | 100 ℃ | 0.910 | 26.18 | 72.95 | 17.38 |
|  | 120 ℃ | 0.896 | 26.13 | 71.24 | 16.69 |


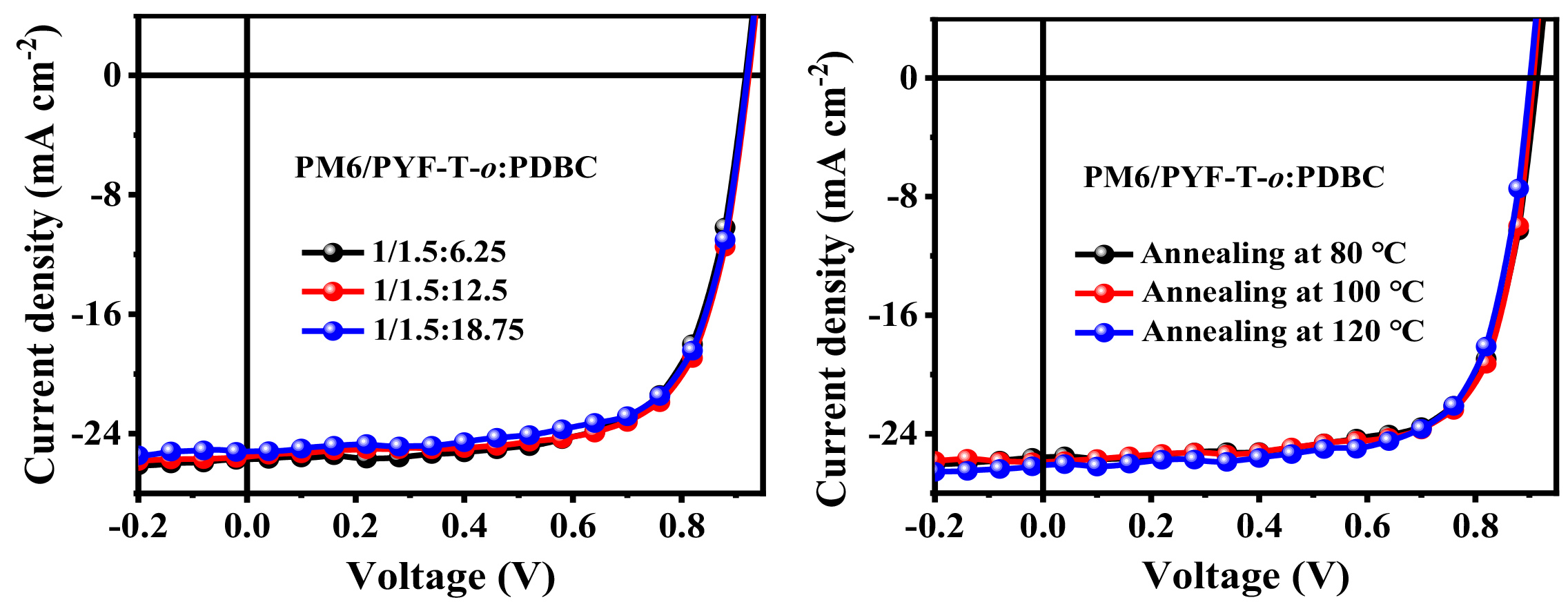


**Fig. S13**. *J−V* curves of PM6/PYF-T-*o*:PDBC devices casted by SqP method without or with thermal annealing treatment.

**Table S6** Photovoltaic parameters based on PM6/PYF-T-*o*:PDBC devices casted by SqP method with different treatment

| PM6/PYF-T-*o*:PDBC | Annealing | *V*_OC_ [V] | *J*_SC_ [mA cm^−2^] | FF [%] | PCE_max_ [%] |
| --- | --- | --- | --- | --- | --- |
| 1/1.5:6.25 | No | 0.918 | 25.81 | 68.97 | 16.34 |
| 1/1.5:12.5 | No | 0.923 | 25.64 | 70.35 | 16.65 |
| 1/1.5:18.75 | No | 0.924 | 25.23 | 69.87 | 16.30 |
| 1/1.5:12.5 | 80 ℃ | 0.914 | 25.52 | 71.66 | 16.72 |
|  | 100 ℃ | 0.908 | 25.91 | 72.25 | 17.01 |
|  | 120 ℃ | 0.902 | 26.06 | 71.37 | 16.77 |


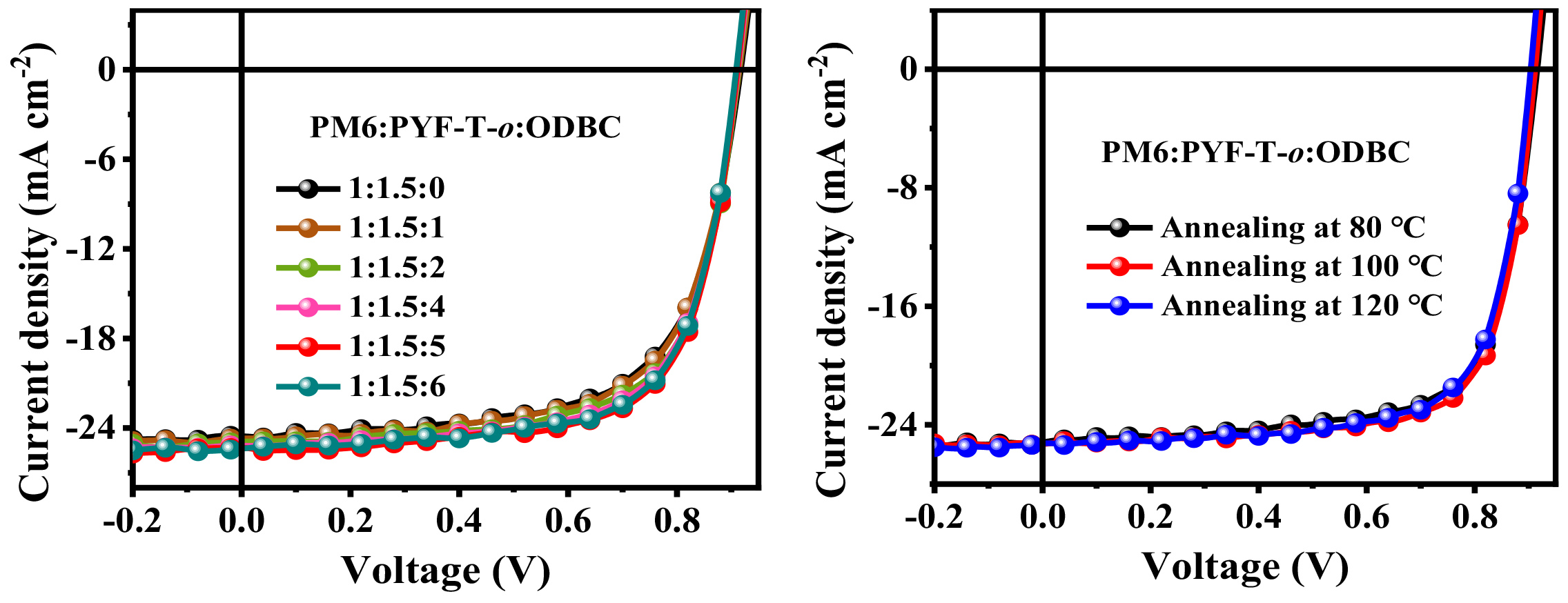


**Fig. S14** *J−V* curves of PM6:PYF-T-*o*:ODBC devices fabricated by blend-casting method and chloroform solvent without or with thermal annealing treatment

**Table S7** Photovoltaic parameters based on PM6:PYF-T-*o*:ODBC devices fabricated by blend-casting method and chloroform solvent with different treatment

| PM6:PYF-T-*o*:ODBC | Annealing | *V*_OC_ [V] | *J*_SC_ [mA cm^−2^] | FF [%] | PCE_max_ [%] |
| --- | --- | --- | --- | --- | --- |
| 1:1.5:0 | No | 0.917 | 24.55 | 65.44 | 14.73 |
| 1:1.5:1 | No | 0.914 | 24.70 | 65.95 | 14.90 |
| 1:1.5:2 | No | 0.913 | 24.92 | 67.87 | 15.45 |
| 1:1.5:4 | No | 0.912 | 25.18 | 68.16 | 15.66 |
| 1:1.5:5 | No | 0.911 | 25.32 | 69.60 | 16.05 |
| 1:1.5:6 | No | 0.909 | 25.39 | 68.76 | 15.86 |
| 1:1.5:5 | 80 ℃ | 0.916 | 25.20 | 70.68 | 16.31 |
|  | 100 ℃ | 0.911 | 25.31 | 72.87 | 16.81 |
|  | 120 ℃ | 0.904 | 25.28 | 71.55 | 16.33 |


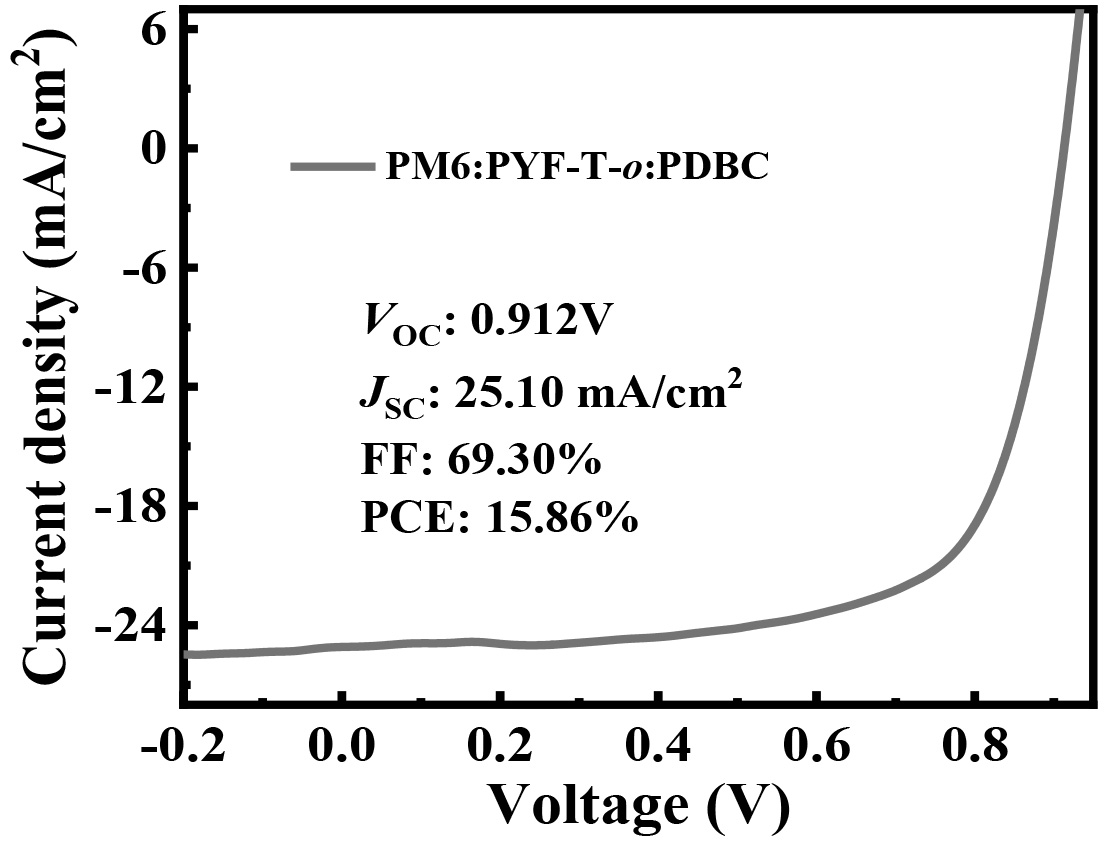


**Fig. S15** *J-V* curves of PM6:PYF-T-*o*:PDBC devices casted by chloroform solvent


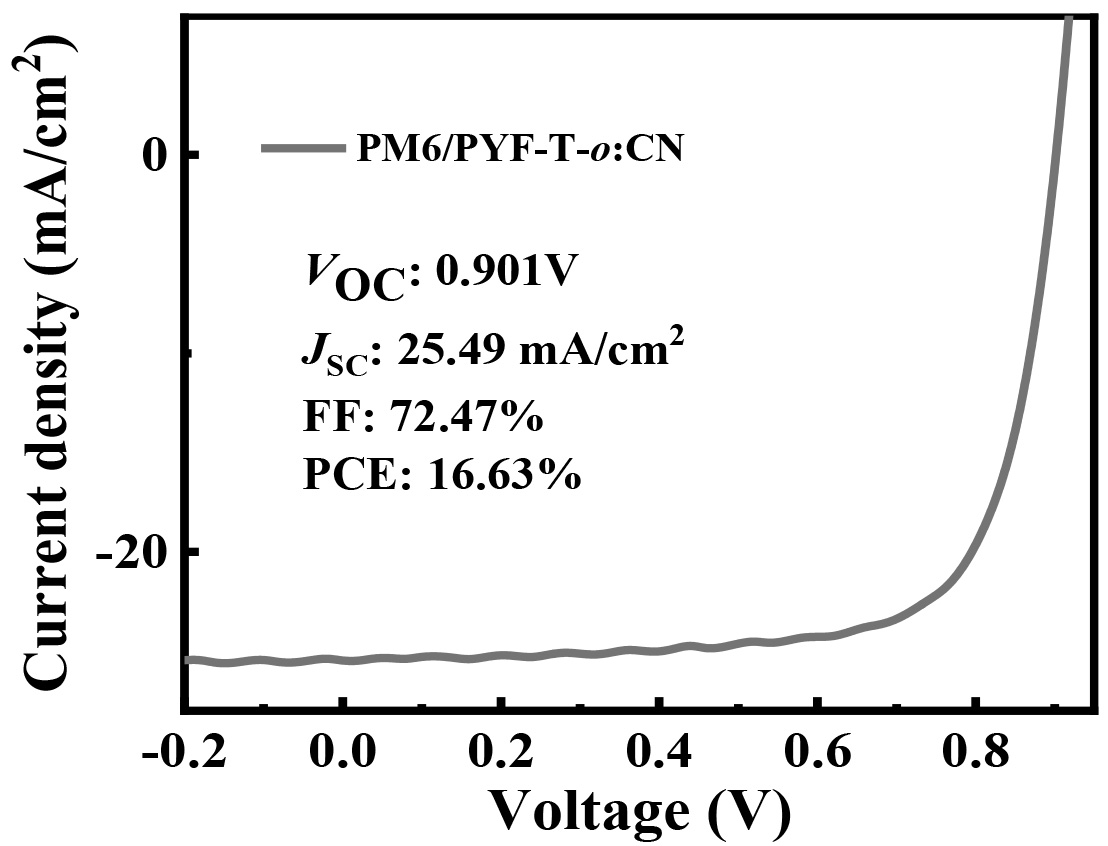


**Fig. S16** *J−V* curves of toluene-processed PM6/PYF-T-*o*:CN devices casted by SqP method.


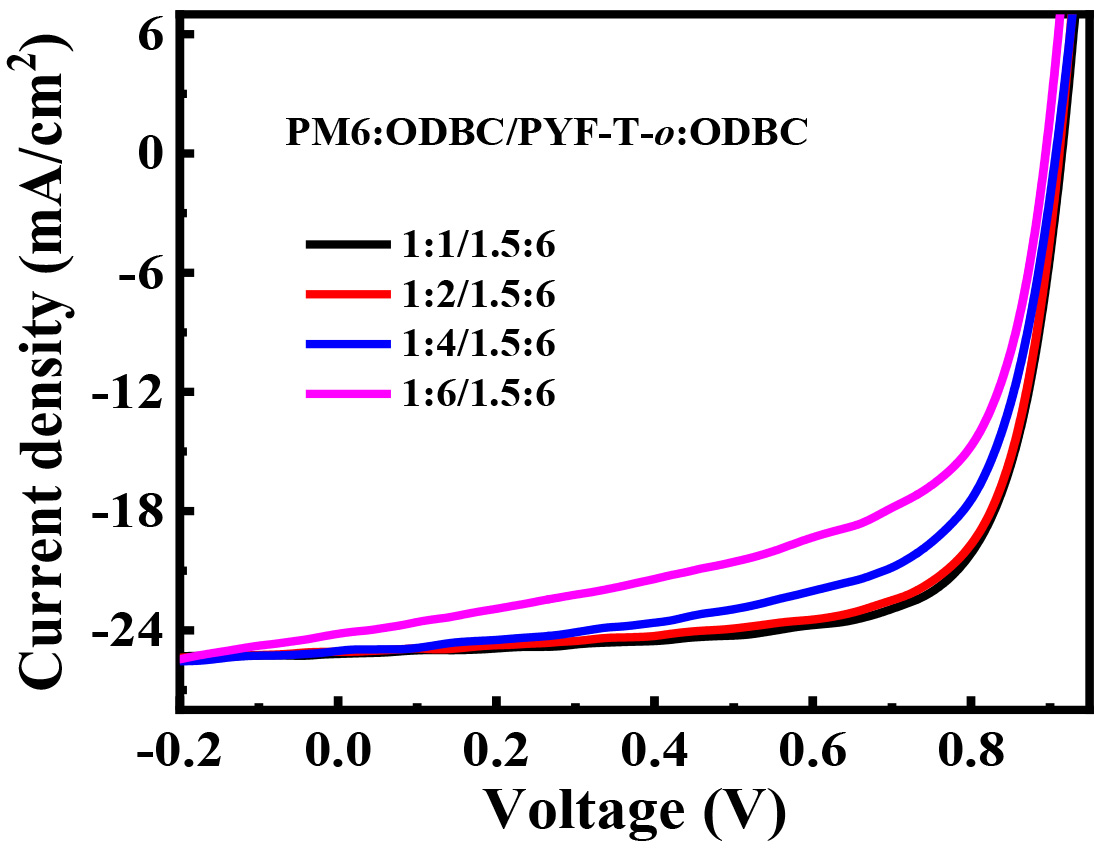


**Fig. S17** *J−V* curves of PM6:ODBC/PYF-T-*o*:ODBC devices fabricated by SqP method

**Table S8** Photovoltaic performance parameters based on PM6:ODBC/PYF-T-*o*:ODBC device fabricated by SqP method

| PM6:ODBC/PYF-T-*o*:ODBC | *V*_OC_ [V] | *J*_SC_ [mA cm^−2^] | FF [%] | PCE_max_ [%] |
| --- | --- | --- | --- | --- |
| 1:1/1.5:6 | 0.913 | 25.18 | 71.90 | 16.53 |
| 1:2/1.5:6 | 0.910 | 25.08 | 70.76 | 16.15 |
| 1:4/1.5:6 | 0.906 | 25.02 | 64.76 | 14.68 |
| 1:6/1.5:6 | 0.892 | 24.16 | 58.16 | 12.54 |


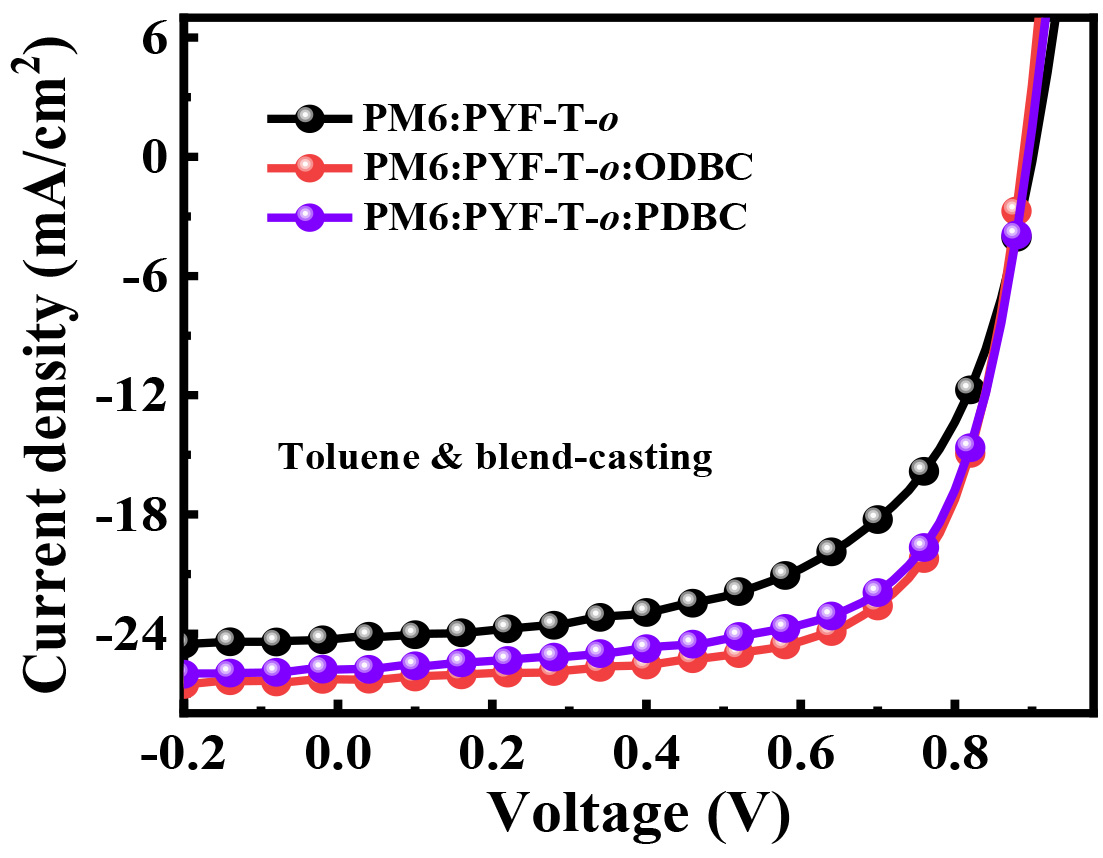


**Fig. S18** *J−V* characteristics of toluene-processed PM6:PYF-T-*o*:additives devices casted by blend-casting method

**Table S9** Photovoltaic parameters for toluene-processed PM6:PYF-T-*o* devices casted by the common blend-casting method

| Active layer | *V*_OC_ (V) | *J*_SC_ (mA cm^-2^) | FF (%) | PCE (%) |
| --- | --- | --- | --- | --- |
| PM6:PYF-T-*o* | 0.900 | 24.25 | 58.70 | 12.81 |
| PM6:PYF-T-*o*:ODBC | 0.887 | 26.29 | 67.66 | 15.78 |
| PM6:PYF-T-*o*:PDBC | 0.894 | 25.80 | 66.48 | 15.33 |


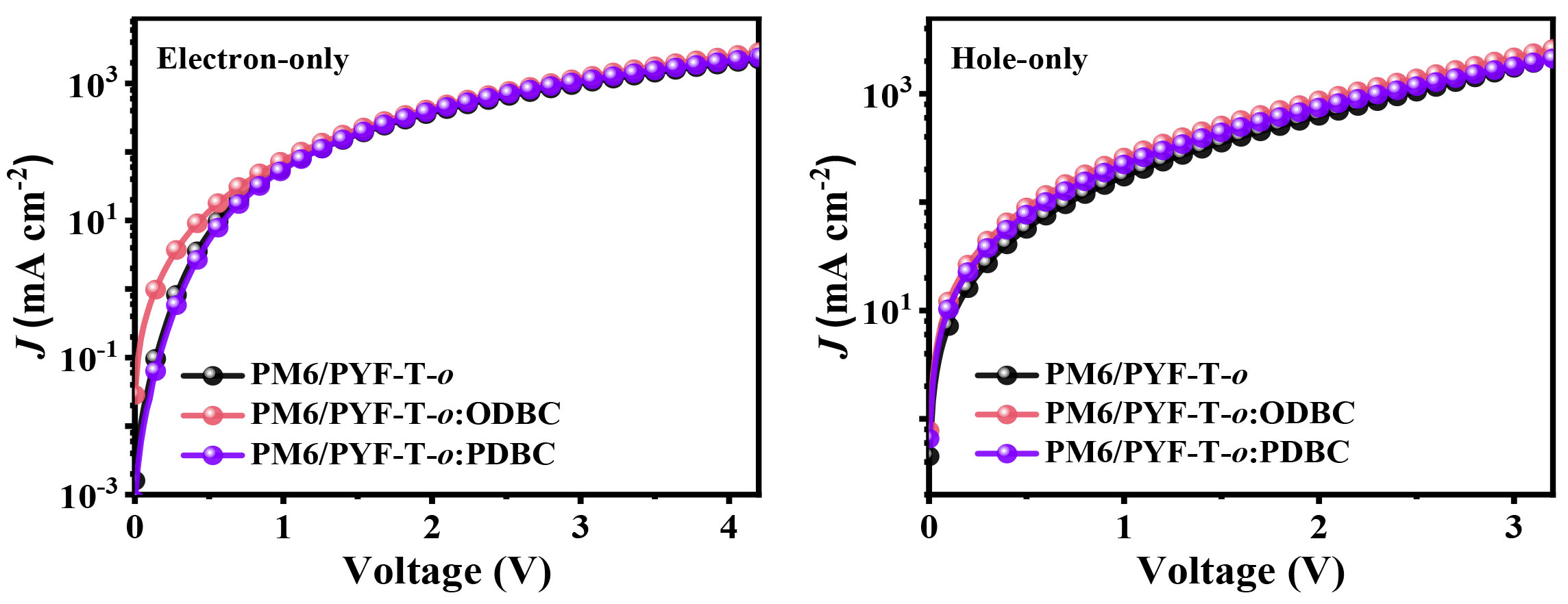


**Fig. S19** *J−V* curves of left) electron-only (ITO/ZnO/PM6/PYF-T-*o*:additive /PNDIT-F3N/Ag) and right) hole-only (ITO/PEDOT:PSS/PM6/PYF-T-*o*:additive/MoO_3_/Ag) devices based on SqP system cast by toluene solvent


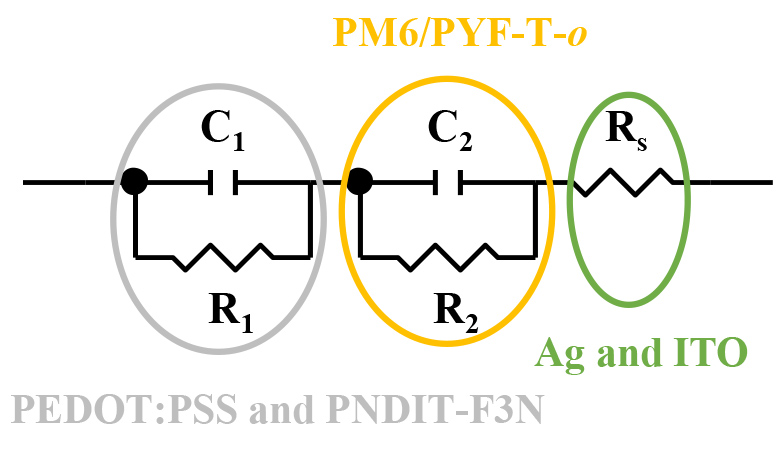


**Fig. S20** The equivalent circuit for fitting impedance spectroscopy. R_s_ corresponds to the electrode resistance including ITO and Ag. Parallel R_1_ and C_1_ correspond to the bulk resistance and capacitance of the PM6/PYF-T-*o* layer. Parallel R_2_ and C_2_ correspond to resistance and capacitance of the PEDOT:PSS and PNDIT-F3N interlayer

**Table S10** Fitting Parameters of impedance spectroscopy using equivalent circuit

| Active layer | R_s_ [Ω] | C_1_ [nF] | R_1_ [Ω] | C_2_ [nF] | R_2_ [Ω] |
| --- | --- | --- | --- | --- | --- |
| PM6/PYF-T-*o* | 32.03 | 1.89 | 1450 | 14.8 | 451.1 |
| PM6/PYF-T-*o*:ODBC | 37.50 | 4.37 | 1218 | 7.10 | 157.5 |
| PM6/PYF-T-*o*:PDBC | 38.11 | 4.51 | 1358 | 7.52 | 270.5 |


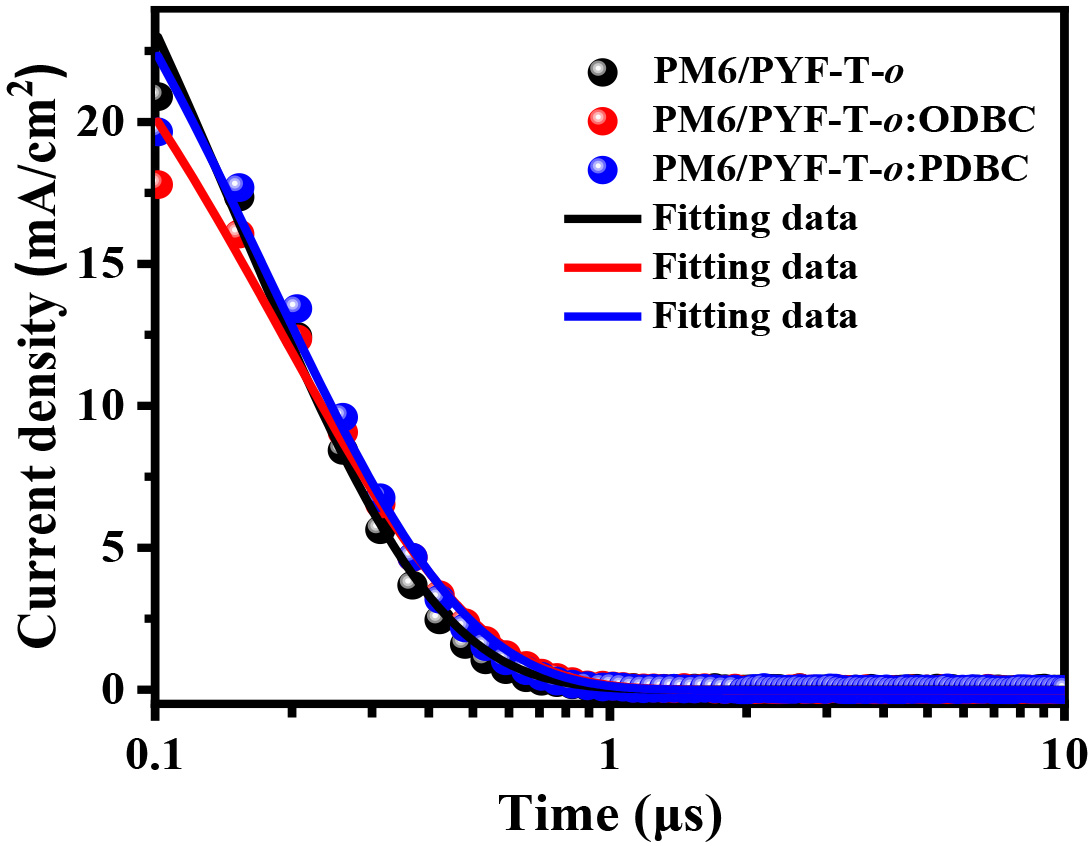


**Fig. S21** The trapped defect state volume density *N*_t_ of PM6/PYF-T-*o*:additives devices


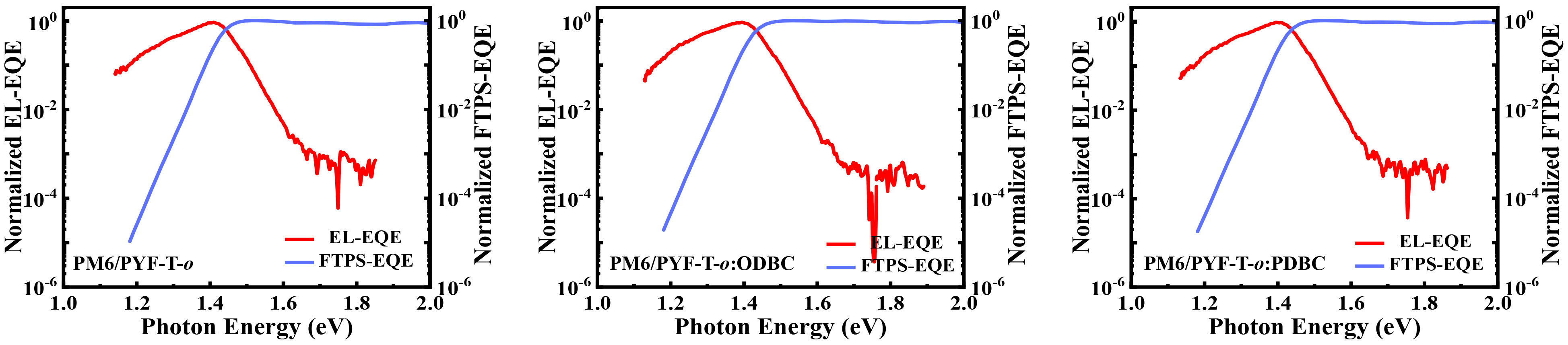


**Fig. S22** Semi-logarithmic plots of normalized EL and measured EQE by FTPS as a function of energy for devices based on PM6/PYF-T-*o*, PM6/PYF-T-*o*:ODBC and PM6/PYF-T-*o*:PDBC devices

**Table S11** Detailed *E*_loss_ parameters in PM6/PYF-T-*o*:additive devices

| Additive | *V*_OC_^a)^ (V) | *E*_g_^b)^ (eV) | *qV*_loss_ (eV) | *V*_OC_ ^SQ c)^  (V) | *V*_OC_ ^rad d)^  (V) | Δ*E*_1_  (eV) | Δ*E*_2_^e)^  (eV) | Δ*E*_3_^f)^  (eV) |
| --- | --- | --- | --- | --- | --- | --- | --- | --- |
| without | 0.920 | 1.436 | 0.516 | 1.173 | 1.128 | 0.263 | 0.045 | 0.208 |
| ODBC | 0.915 | 1.424 | 0.509 | 1.162 | 1.118 | 0.262 | 0.044 | 0.203 |
| PDBC | 0.909 | 1.427 | 0.517 | 1.164 | 1.122 | 0.263 | 0.041 | 0.213 |

a) *V*_OC_: measured in the voltage loss test without an aperture.

b) *E*_g_: optical bandgaps, via the derivatives of the EQE spectra (dEQE/dE).

c) *V*𝑜𝑐^𝑆𝑄^: Schokley-Queisser limit to *V*_OC_.

d) *V*𝑜𝑐^𝑟𝑎𝑑^: radiative limit to *V*oc, measured using EQE_EL_

e) ∆*E*_2_ ((*V*oc^SQ^-*V*oc^rad^): voltage losses due to non-ideal absorption (it was calculated from EL and FTPS measurements).

f) ∆*E*_3_ (∆𝑉𝑜𝑐 ^𝑛𝑜𝑛−𝑟𝑎𝑑^): voltage losses due to non-radiative recombination only.


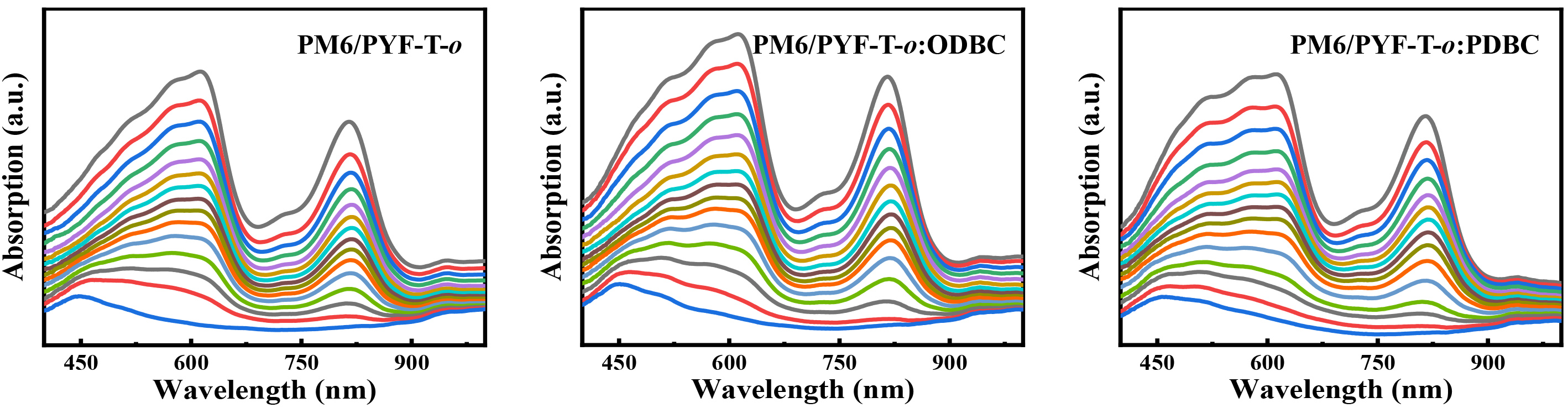


**Fig. S23** Depth-dependent absorption spectra of three PM6/PYF-T-*o*:additive films under etching time


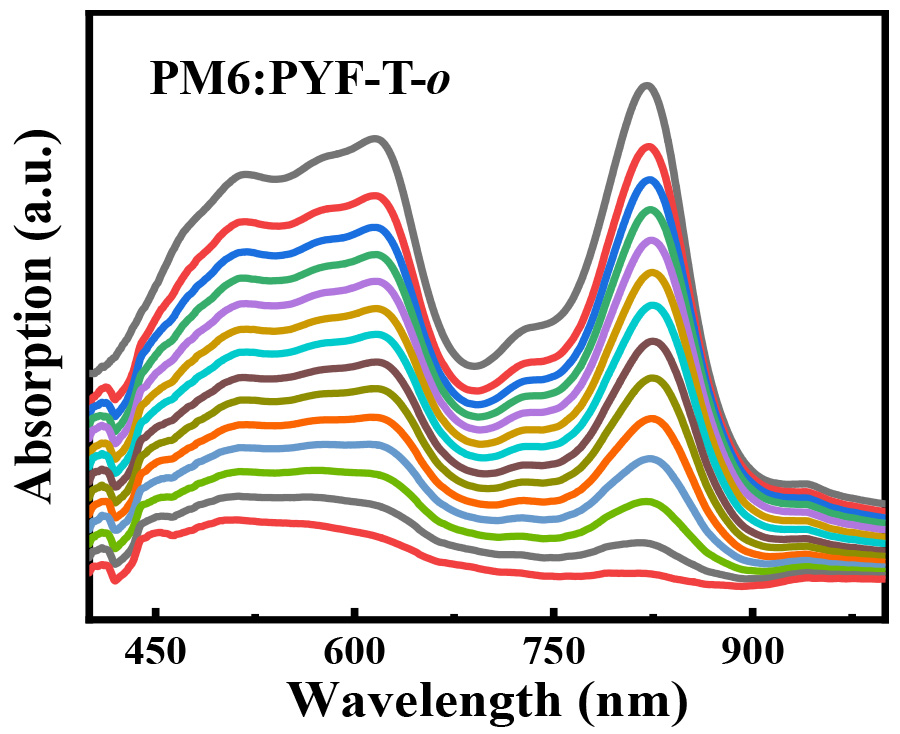


**Fig. S24** Depth-dependent absorption spectra of blending-casted PM6:PYF-T-*o* films under etching time


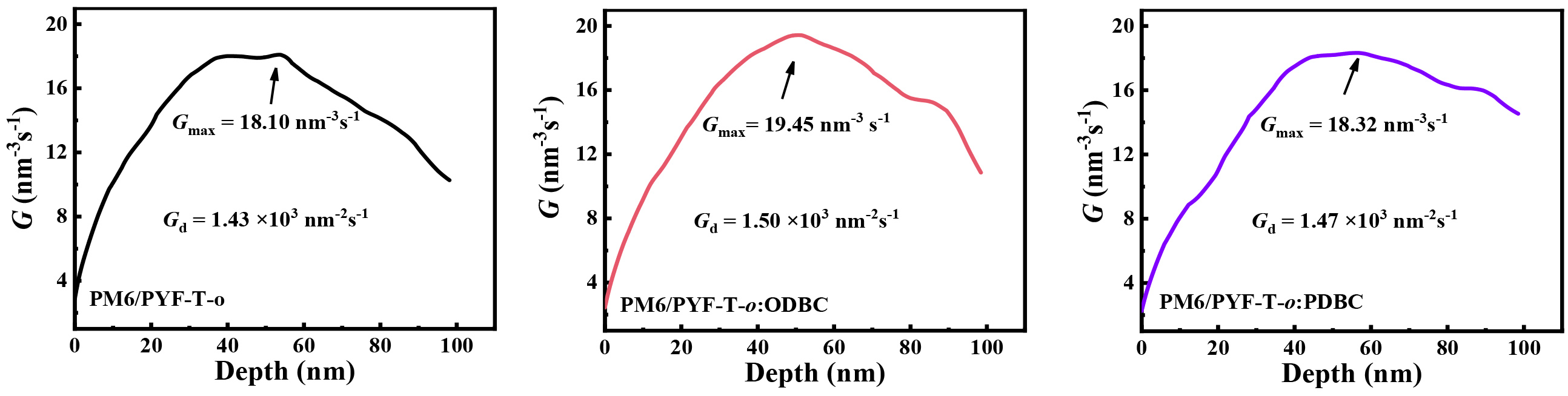


**Fig. S25** The calculated exciton generation rate curves on the depth of active layer film


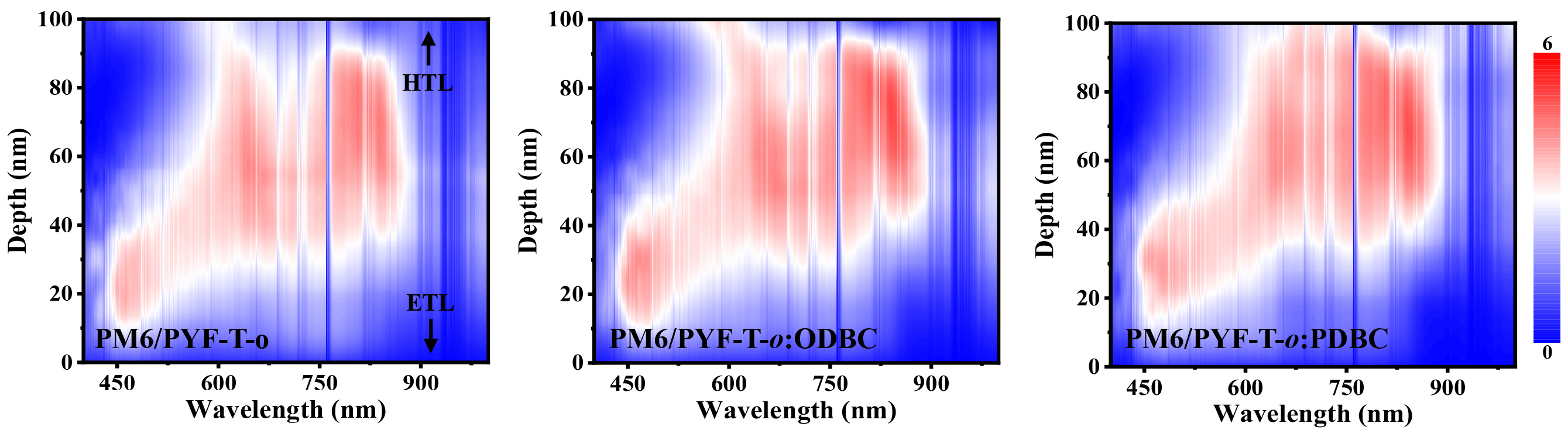


**Fig. S26** The exciton generation cloud maps in PM6/PTF-T-*o* films with depth direction, the unit of scale bar is 10^25^ m^−3^nm^−1^s^−1^


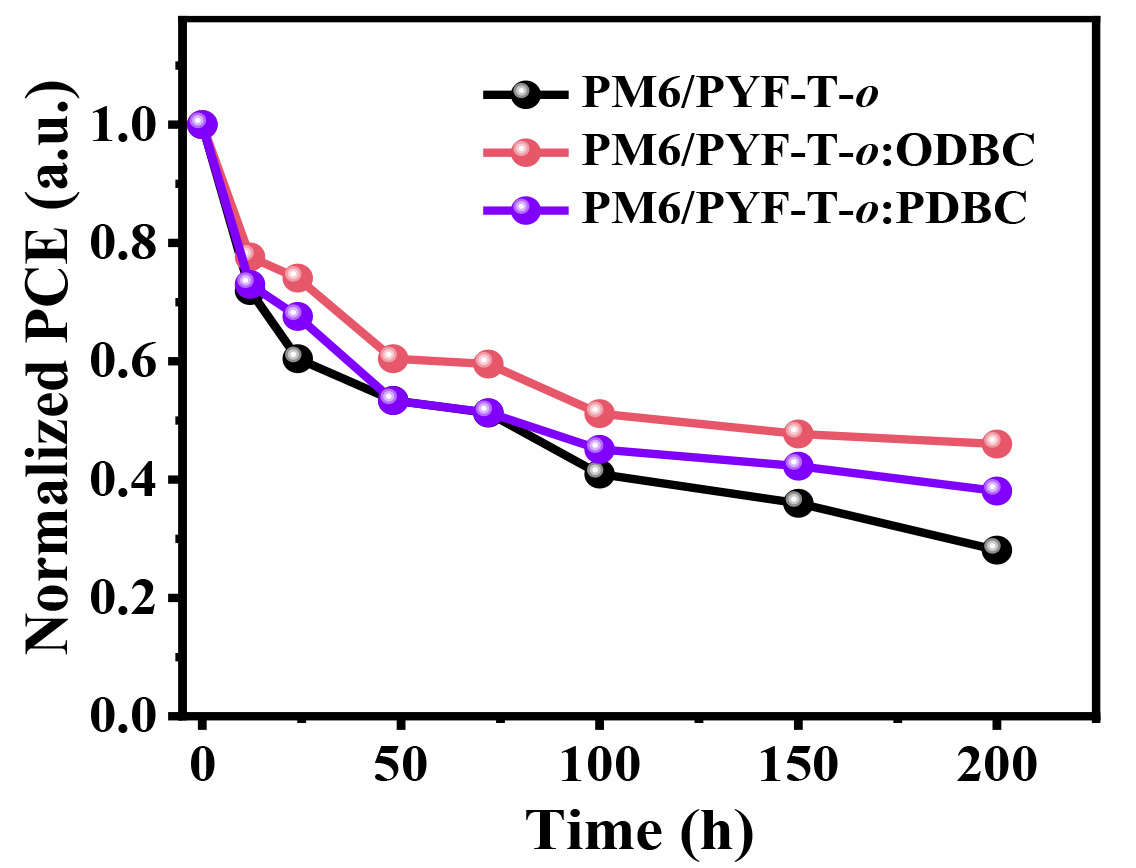


**Fig. S27** Normalized PCE of SqP device with different additives as a function of annealing time at 80 °C


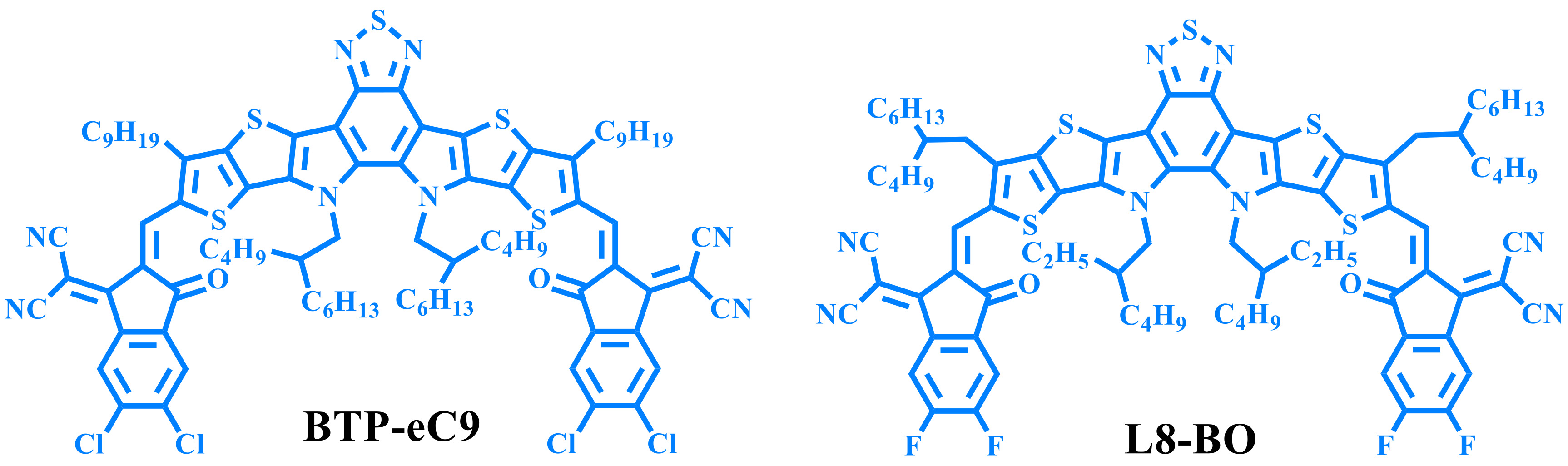


**Fig. S28** The chemical structures of BTP-eC9 and L8-BO acceptors


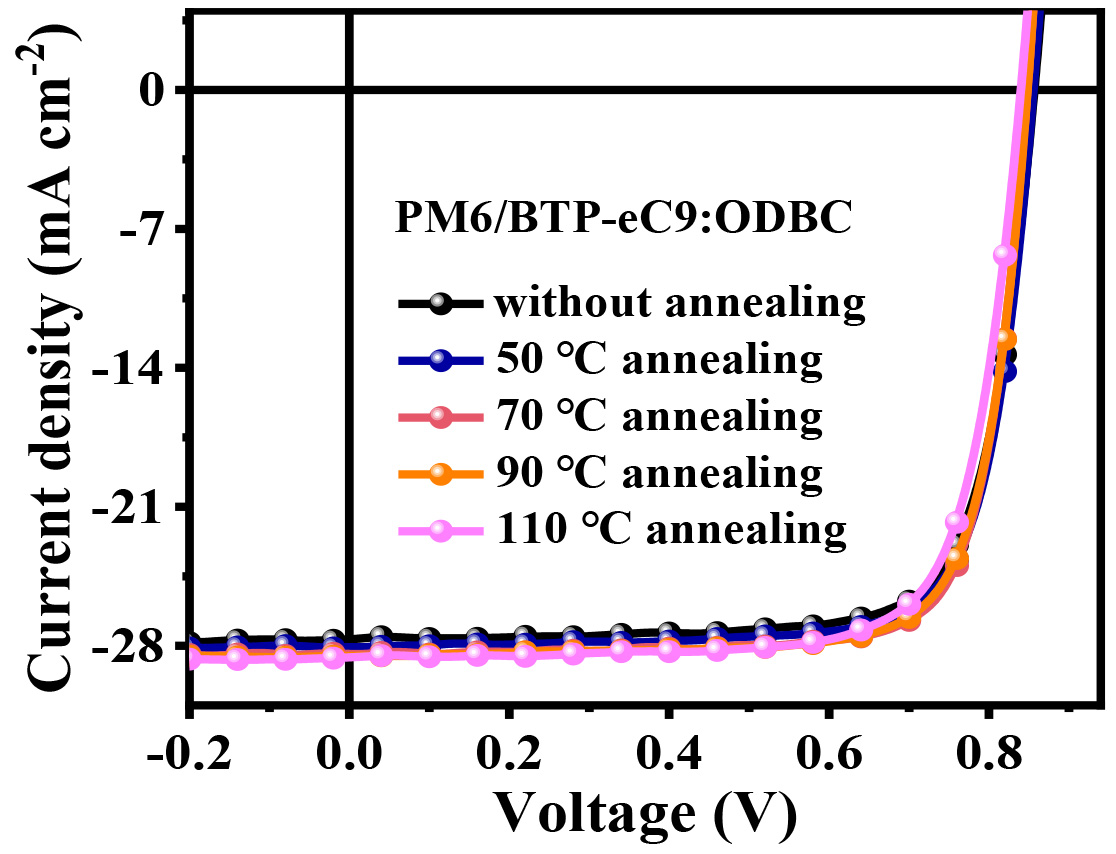


**Fig. S29** *J−V* curves of PM6/BTP-eC9:ODBC devices casted by SqP method with thermal annealing treatment, where PEDOT:PSS was used hole transport layer

**Table S12** Photovoltaic parameters based on PM6/BTP-eC9:ODBC devices casted by SqP method with different annealing temperature

| PM6/BTP-eC9:ODBC | Annealing | V_OC_ [V] | J_SC_ [mA cm^−2^] | FF [%] | PCE_max_ [%] |
| --- | --- | --- | --- | --- | --- |
| 1/1:2 | 0 | 0.856 | 27.68 | 76.24 | 18.06 |
|  | 50 | 0.854 | 28.06 | 76.75 | 18.39 |
|  | 70 | 0.847 | 28.41 | 78.20 | 18.81 |
|  | 90 | 0.846 | 28.51 | 77.16 | 18.61 |
|  | 110 | 0.839 | 28.58 | 75.38 | 18.07 |


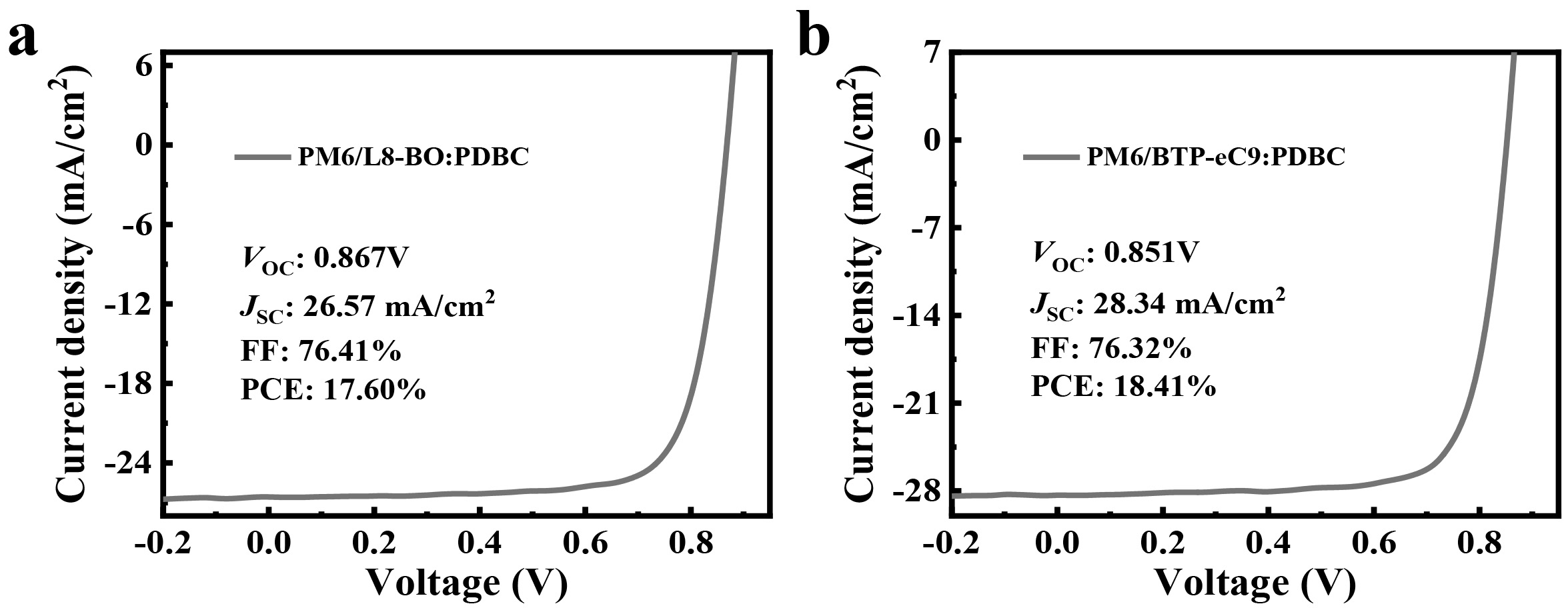


**Fig. S30** *J−V* curves of toluene -processed SqP devices casted by PEDOT:PSS hole transport layer for a) PM6/L8-BO:PDBC and b) PM6/BTP-eC9:PDBC


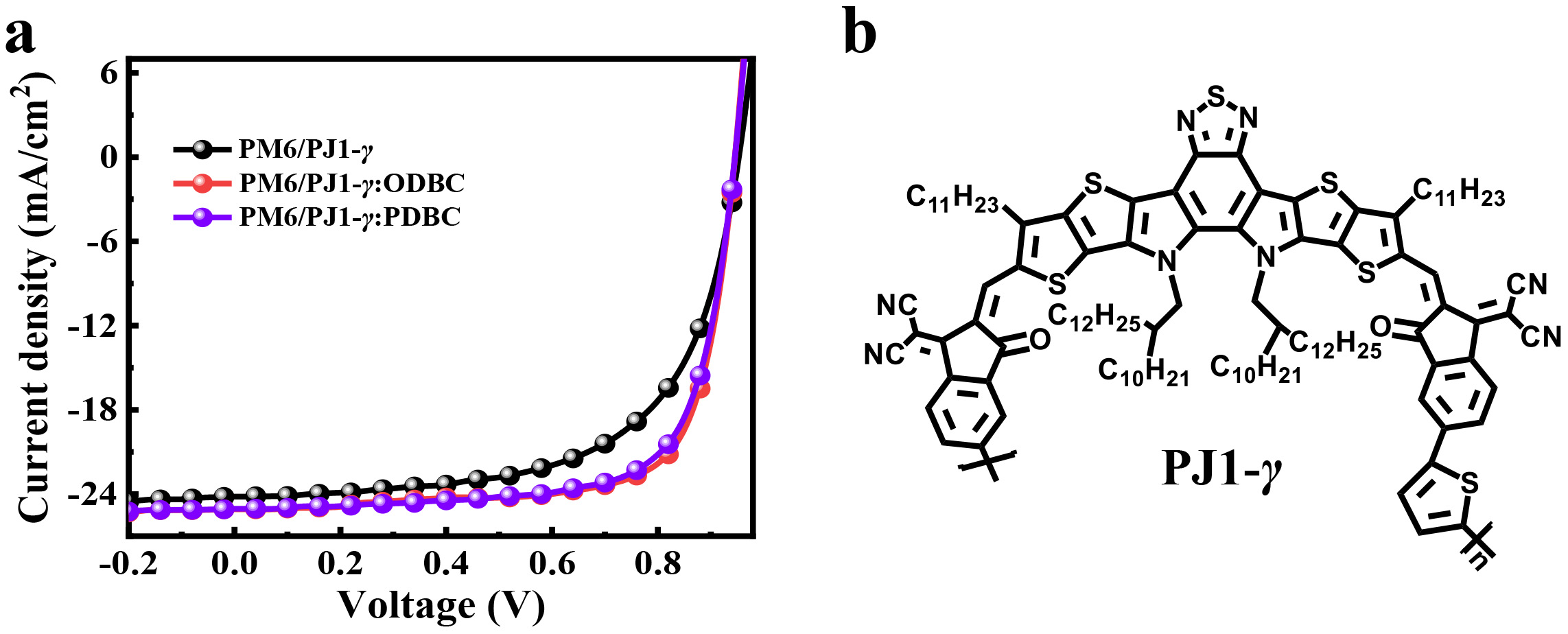


**Fig. S31** **a**) *J−V* characteristics of toluene-processed PM6/PJ1-*γ* devices using either PEDOT:PSS as the hole transport layer. **b**) The chemical structure of PJ1-*γ*

**Table S13** Photovoltaic parameters for PM6/PJ1-*γ* devices using toluene as the main solvent

| Active layer | *V*_OC_ (V) | *J*_SC_ (mA cm^-2^) | FF (%) | PCE (%) |
| --- | --- | --- | --- | --- |
| PM6/PJ1-*γ* | 0.952 | 24.17 | 62.39 | 14.36 |
| PM6/PJ1-*γ*:ODBC | 0.944 | 25.08 | 73.57 | 17.41 |
| PM6/PJ1-*γ*:PDBC | 0.944 | 25.02 | 71.76 | 16.93 |


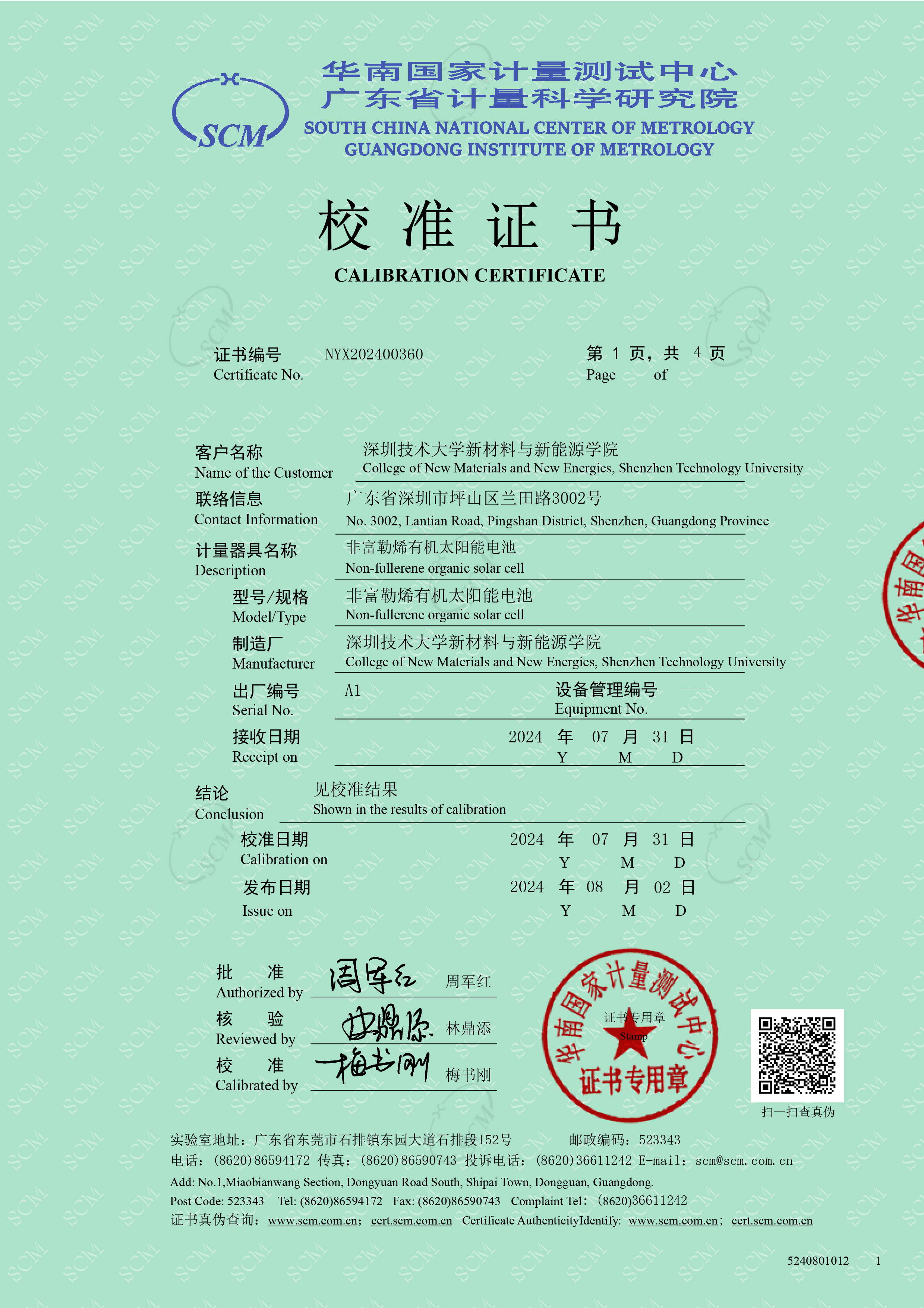


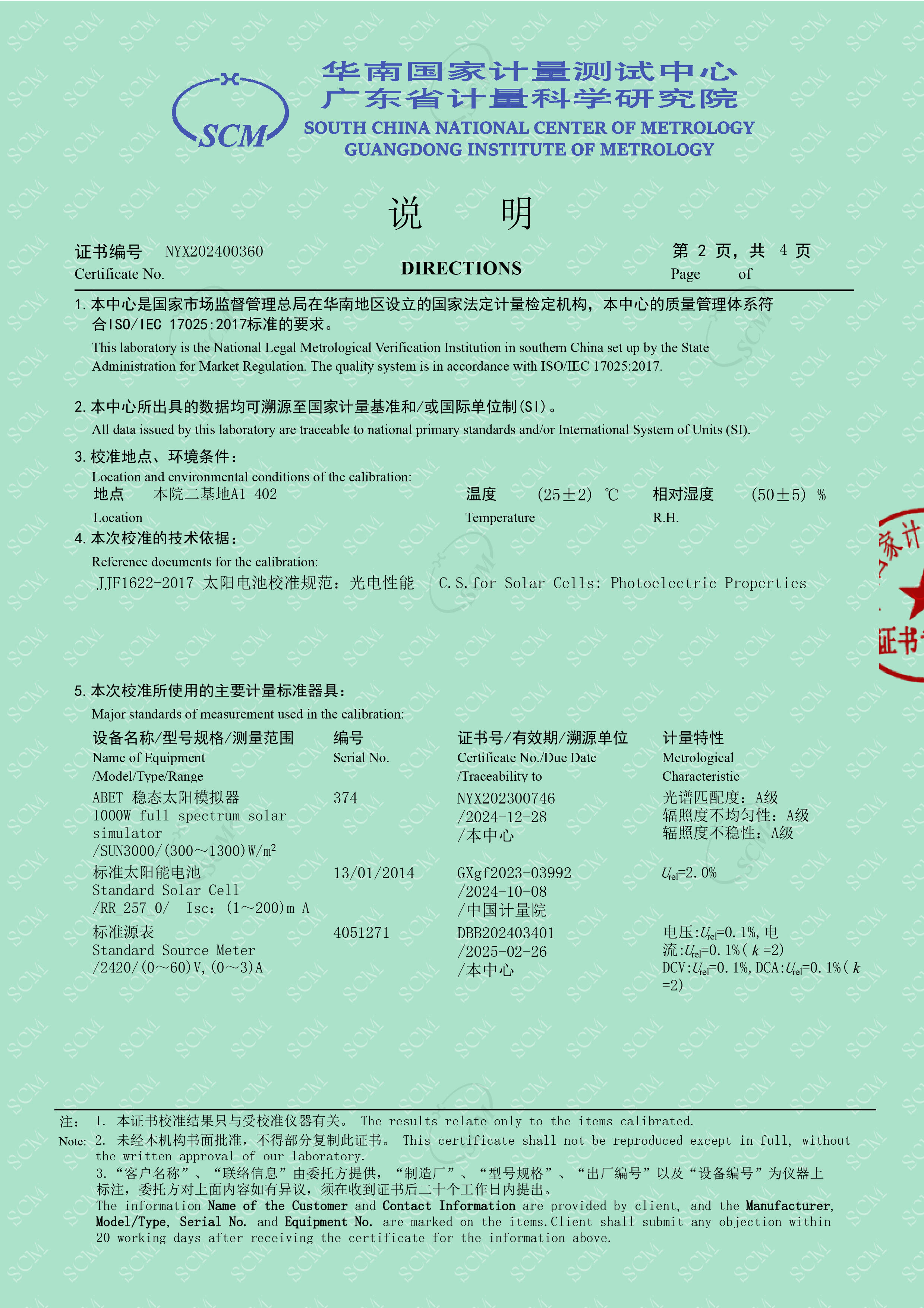

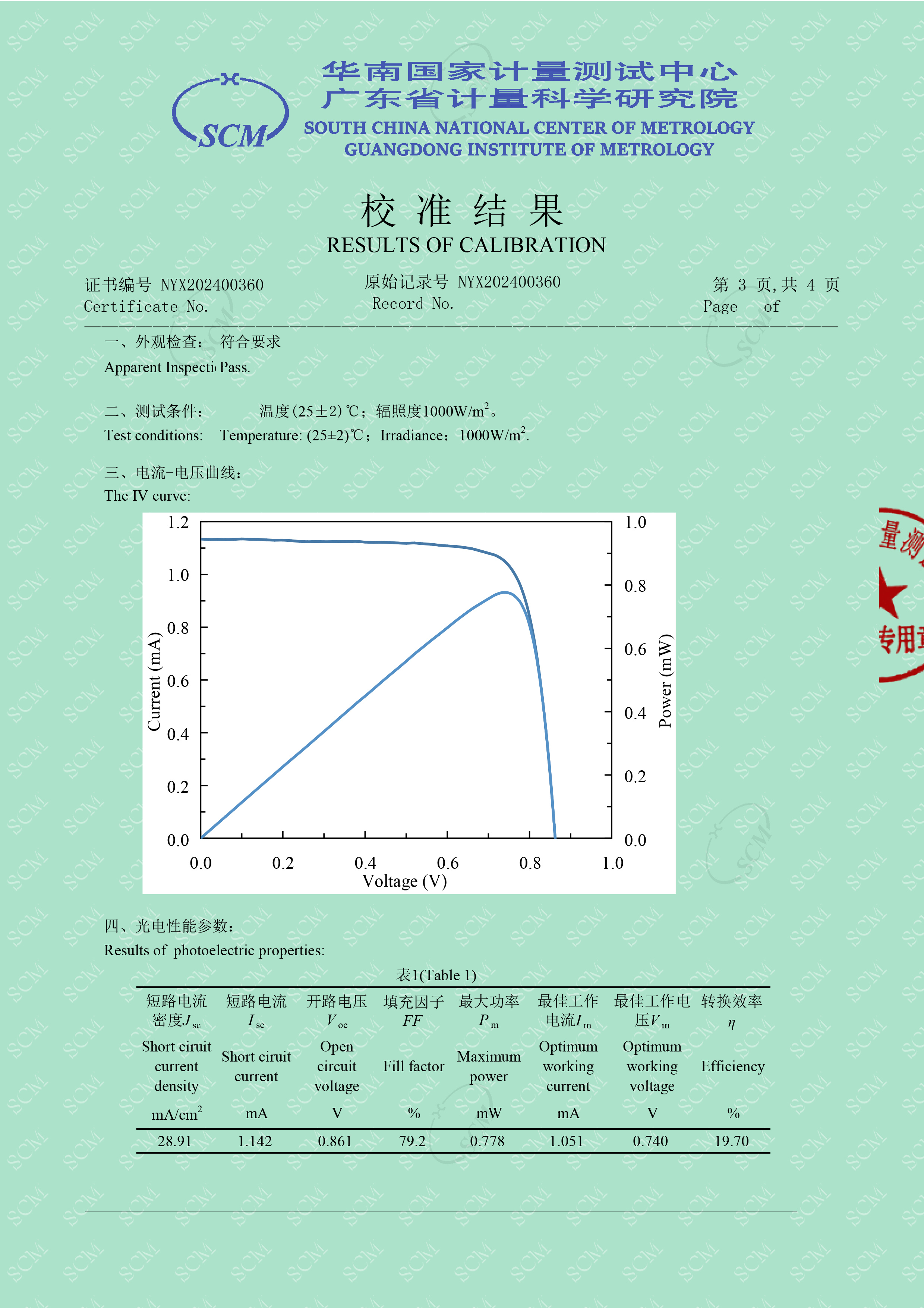

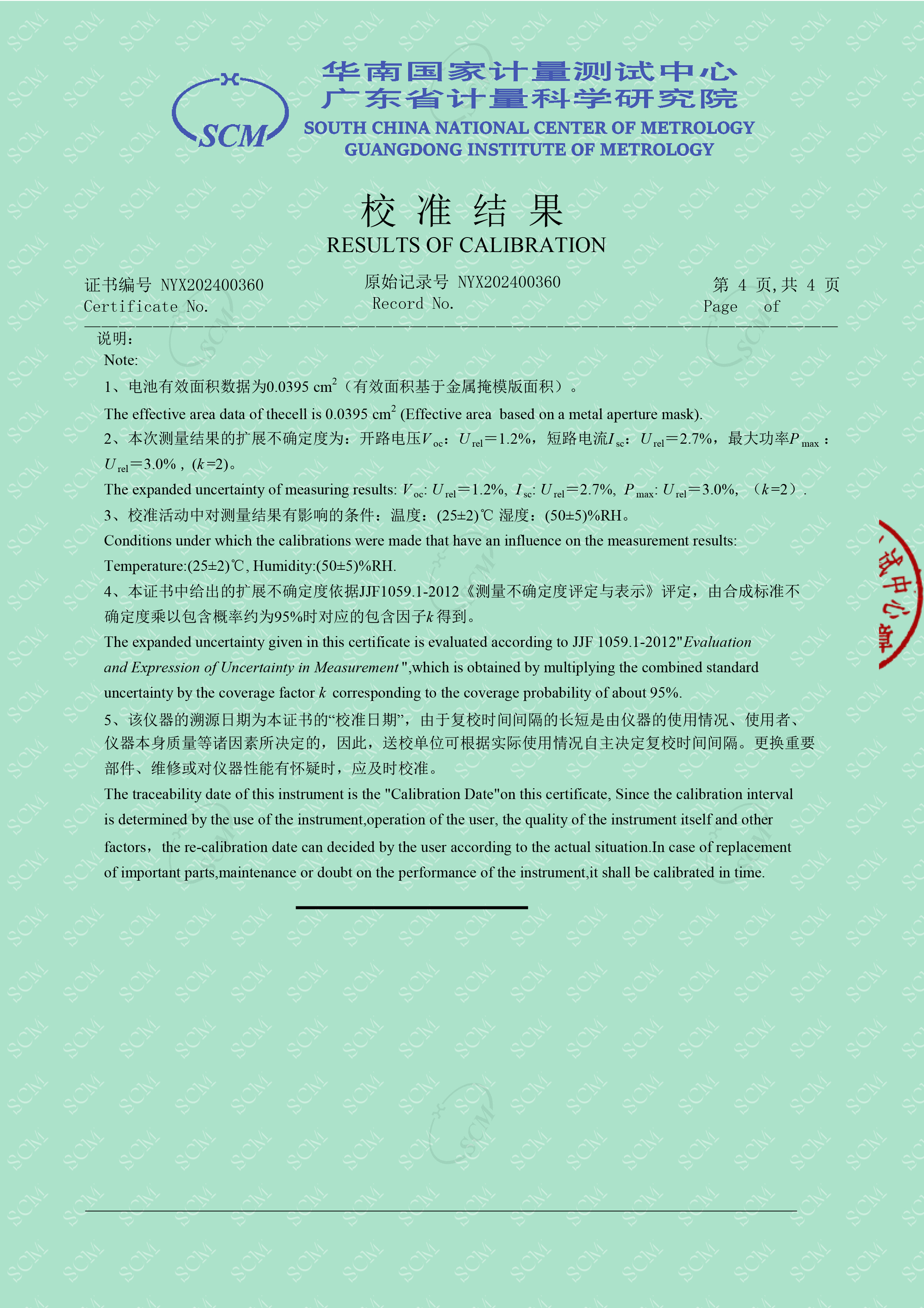


**Fig. S32** Certificate of the optimal device issued by South China National Center of Metrology, China

**Table S14** Summary of the recent progress of binary OSCs processed from non-halogenated solvent with PCEs of over 17%

| Active layer | Solvent | *V*_OC_ [V] | *J*_SC_ [mA cm^-2^] | FF [%] | PCE [%] | References |
| --- | --- | --- | --- | --- | --- | --- |
| D18:DTC11 | CS_2_/*o*-XY | 0.858 | 27.5 | 80.5 | 19.0 | [S3] |
| PM6:G-Trimer | *o*-XY/1-CN | 0.896 | 25.75 | 79.3 | 19.01 | [S4] |
| PM6:Y6-HU | *o*-XY/DBrB | 0.87 | 27 | 79.0 | 18.6 | [S5] |
| PTF5:Y6-BO | *o*-XY | 0.84 | 27 | 77.2 | 18.2 | [S6] |
| PM6:EV-i | *o*-XY | 0.897 | 26.6 | 76.56 | 18.27 | [S7] |
| D18-Cl:L8-BO-x | TL/DTT | 0.893 | 26.78 | 79.6 | 19.04 | [S8] |
| PM6:L15 | *o*-XY/DTT-2 | 0.93 | 25.95 | 77.26 | 18.72 | [S9] |
| PM6:L8-Ph | *o*-XY/DTT | 0.87 | 26.4 | 80.41 | 18.4 | [S10] |
| PBQ6:PYF-T-*o* | TL | 0.886 | 25.12 | 76.64 | 17.06 | [S4] |
| PM6:G-Trimer | *o*-XY/1-CN | 0.911 | 25.4 | 79.49 | 18.39 | [S11] |
| PM6:BO-4Cl | *o*-XY/DIO | 0.841 | 26.73 | 79 | 17.67 | [S12] |
| PM6:BTP-eC9 | *o*-XY/DIO | 0.84 | 26.65 | 78.1 | 17.48 | [S13] |
| PM6:BTP-SO-2F | *o*-XY/DIO | 0.909 | 24.54 | 78.9 | 17.6 | [S14] |
| PM6:PY-DT | *o*-XY/2-MN | 0.954 | 23.09 | 77.3 | 17.03 | [S15] |
| PM6:BTP-BO-4Cl | TL/BV | 0.854 | 26.1 | 77.7 | 17.33 | [S16] |
| PM6:BTP-eC9 | *o*-XY/CS_2_ | 0.85 | 26.2 | 78.9 | 17.6 | [S17] |
| PM6:BTP-eC9 | *o*-XY/1-CN | 0.84 | 26.77 | 76.33 | 17.15 | [S18] |
| PM6:eC11 | *o*-XY/PN | 0.846 | 26.23 | 77.3 | 17.15 | [S19] |
| PM6:CH7 | *o*-XY/DIB | 0.881 | 25.82 | 76.88 | 17.49 | [S20] |
| PM6:Y6-HU | *o*-XY/DPE | 0.851 | 26.68 | 76.52 | 17.38 | [S21] |
| PM6:Y6-HU | *o*-XY | 0.87 | 25.6 | 77.9 | 17.4 | [S10] |
| D18:L8-BO | CS_2_/PX | 0.885 | 26.25 | 75.3 | 17.5 | [S22] |
| PM6/PYF-T-*o* | TL/ODBC | 0.910 | 26.18 | 72.95 | 17.38 | This work |
| PM6/BTP-eC9 | TL/ODBC | 0.861 | 28.89 | 80.41 | 20.00 | This work |


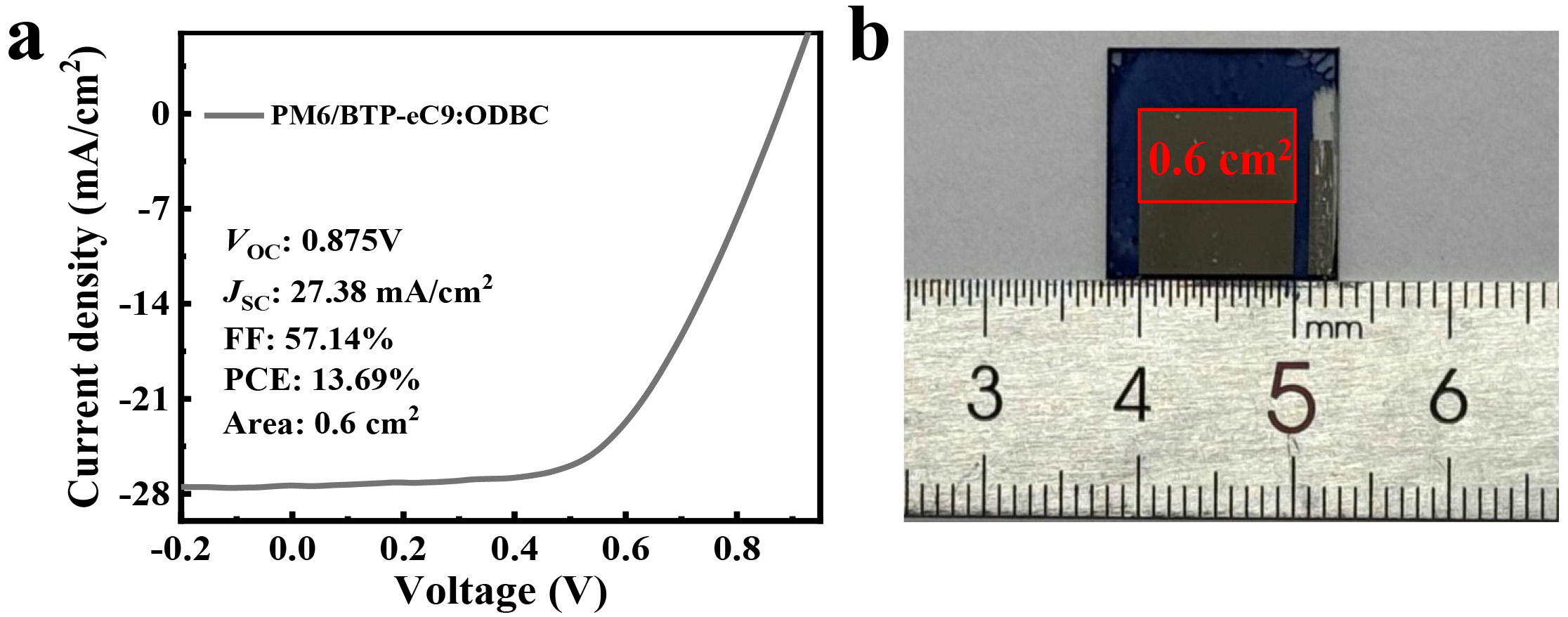


**Fig. S33 a**) The *J-V* curve and **b**) the actual images of PM6/L8-BO:ODBC devices with 0.6 cm^2^ area

**Supplementary References**

1. G. Juska, K. Arlauskas, M. Viliunas, J. Kocka, Extraction current transients: new method of study of charge transport in microcrystalline silicon. Phys. Rev. Lett. **84**(21), 4946–4949 (2000). <https://doi.org/10.1103/PhysRevLett.84.4946>
2. L. Yan, Z. Liang, J. Si, P. Gong, Y. Wang et al., Ultrafast kinetics of chlorinated polymer donors: a faster excitonic dissociation path. ACS Appl. Mater. Interfaces **14**(5), 6945–6957 (2022). <https://doi.org/10.1021/acsami.1c24348>
3. Z. Zhong, S. Chen, J. Zhao, J. Xie, K. Zhang et al., Non-halogen solvent processed binary organic solar cells with efficiency of 19% and module efficiency over 15% enabled by asymmetric alkyl chain engineering. Adv. Energy Mater. **13**(39), 2302273 (2023). <https://doi.org/10.1002/aenm.202302273>
4. C. Wang, X. Ma, Y.-F. Shen, D. Deng, H. Zhang et al., Unique assembly of giant star-shaped trimer enables non-halogen solvent-fabricated, thermal stable, and efficient organic solar cells. Joule **7**(10), 2386–2401 (2023). <https://doi.org/10.1016/j.joule.2023.09.001>
5. M. Haris, R. Dh, Z. Ullah, Morphological modulation enabled by non-halogenated solvent-processed simple solid additives for high-efficiency organic solar cells. EcoMat **6**(3), e12436 (2024). <https://doi.org/10.1002/eom2.12436>
6. Z.U. Rehman, M. Haris, S.U. Ryu, M. Jahankhan, C.E. Song et al., Trifluoromethyl-substituted conjugated random terpolymers enable high-performance small and large-area organic solar cells using halogen-free solvent. Adv. Sci. **10**(24), e2302376 (2023). <https://doi.org/10.1002/advs.202302376>
7. H. Zhuo, X. Li, J. Zhang, S. Qin, J. Guo et al., Giant molecule acceptor enables highly efficient organic solar cells processed using non-halogenated solvent. Angew. Chem. Int. Ed. **62**(26), e202303551 (2023). <https://doi.org/10.1002/anie.202303551>
8. S. Luo, C. Li, J. Zhang, X. Zou, H. Zhao et al., Auxiliary sequential deposition enables 19%-efficiency organic solar cells processed from halogen-free solvents. Nat. Commun. **14**(1), 6964 (2023). <https://doi.org/10.1038/s41467-023-41978-0>
9. B. Liu, W. Xu, R. Ma, J.-W. Lee, T.A. Dela Peña et al., Isomerized green solid additive engineering for thermally stable and eco-friendly all-polymer solar cells with approaching 19% efficiency. Adv. Mater. **35**(49), e2308334 (2023). <https://doi.org/10.1002/adma.202308334>
10. K. Hu, C. Zhu, K. Ding, S. Qin, W. Lai et al., Solid additive tuning of polymer blend morphology enables non-halogenated-solvent all-polymer solar cells with an efficiency of over 17%. Energy Environ. Sci. **15**(10), 4157–4166 (2022). <https://doi.org/10.1039/D2EE01727J>
11. D. Wang, G. Zhou, Y. Li, K. Yan, L. Zhan et al., High-performance organic solar cells from non-halogenated solvents. Adv. Funct. Mater. **32**(4), 2107827 (2022). <https://doi.org/10.1002/adfm.202107827>
12. Y. Zhang, K. Liu, J. Huang, X. Xia, J. Cao et al., Graded bulk-heterojunction enables 17% binary organic solar cells *via* nonhalogenated open air coating. Nat. Commun. **12**(1), 4815 (2021). <https://doi.org/10.1038/s41467-021-25148-8>
13. J. Hai, L. Li, Y. Song, X. Liu, X. Shi et al., Ending group modulation of asymmetric non-fullerene acceptors enables efficient green solvent processed organic solar cells. Chem. Eng. J. **462**, 142178 (2023). <https://doi.org/10.1016/j.cej.2023.142178>
14. J. Song, Y. Li, Y. Cai, R. Zhang, S. Wang et al., Solid additive engineering enables high-efficiency and eco-friendly all-polymer solar cells. Matter **5**(11), 4047–4059 (2022). <https://doi.org/10.1016/j.matt.2022.08.011>
15. X. Xu, L. Yu, H. Yan, R. Li, Q. Peng, Highly efficient non-fullerene organic solar cells enabled by a delayed processing method using a non-halogenated solvent. Energy Environ. Sci. **13**(11), 4381–4388 (2020). <https://doi.org/10.1039/D0EE02034F>
16. X. Song, P. Sun, D. Sun, Y. Xu, Y. Liu et al., Investigation of tunable halogen-free solvent engineering on aggregation and miscibility towards high-performance organic solar cells. Nano Energy **91**, 106678 (2022). <https://doi.org/10.1016/j.nanoen.2021.106678>
17. H. Li, S. Liu, X. Wu, Q. Qi, H. Zhang et al., A general enlarging shear impulse approach to green printing large-area and efficient organic photovoltaics. Energy Environ. Sci. **15**(5), 2130–2138 (2022). <https://doi.org/10.1039/d2ee00639a>
18. R. Ma, C. Yan, P.W. Fong, J. Yu, H. Liu et al., *In situ*and*ex situ*investigations on ternary strategy and co-solvent effects towards high-efficiency organic solar cells. Energy Environ. Sci. **15**(6), 2479–2488 (2022). <https://doi.org/10.1039/d2ee00740a>
19. S. Zhang, H. Chen, P. Wang, S. Li, Z. Li et al., A large area organic solar module with non-halogen solvent treatment, high efficiency, and decent stability. Sol. RRL **7**(7), 2300029 (2023). <https://doi.org/10.1002/solr.202300029>
20. S. Rasool, J.W. Kim, H.W. Cho, Y.J. Kim, D.C. Lee et al., Morphologically controlled efficient air-processed organic solar cells from halogen-free solvent system. Adv. Energy Mater. **13**(7), 2203452 (2023). <https://doi.org/10.1002/aenm.202203452>
21. Z. Abbas, S.U. Ryu, M. Haris, C.E. Song, H.K. Lee et al., Optimized vertical phase separation *via* systematic Y6 inner side-chain modulation for non-halogen solvent processed inverted organic solar cells. Nano Energy **101**, 107574 (2022). <https://doi.org/10.1016/j.nanoen.2022.107574>
22. Y. Su, Z. Ding, R. Zhang, W. Tang, W. Huang et al., High-efficiency organic solar cells processed from a halogen-free solvent system. Sci. China Chem. **66**(8), 2380–2388 (2023). <https://doi.org/10.1007/s11426-023-1608-6>
